# Synthetic4Health: generating annotated synthetic clinical letters

**DOI:** 10.3389/fdgth.2025.1497130

**Published:** 2025-05-30

**Authors:** Libo Ren, Samuel Belkadi, Lifeng Han, Warren Del-Pinto, Goran Nenadic

**Affiliations:** ^1^ Department of Computer Science, University of Manchester, Greater Manchester, Manchester, United Kingdom; ^2^ Department of Engineering, University of Cambridge, Cambridge, United Kingdom; ^3^ Leiden Institute of Advanced Computer Science (LIACS) and Leiden University Medical Center (LUMC), Leiden University, Leiden, Netherlands

**Keywords:** pre-trained language models (PLMs), encoder-only models, encoder–decoder models, named entity recognation, masking and generating, synthetic data creation, clinical NLP (natural language processing)

## Abstract

Clinical letters contain sensitive information, limiting their use in model training, medical research, and education. This study aims to generate reliable, diverse, and de-identified synthetic clinical letters to support these tasks. We investigated multiple pre-trained language models for text masking and generation, focusing on Bio_ClinicalBERT, and applied different masking strategies. Evaluation included qualitative and quantitative assessments, downstream named entity recognition (NER) tasks, and clinically focused evaluations using BioGPT and GPT-3.5-turbo. The experiments show: (1) encoder-only models perform better than encoder–decoder models; (2) models trained on general corpora perform comparably to clinical-domain models if clinical entities are preserved; (3) preserving clinical entities and document structure aligns with the task objectives; (4) Masking strategies have a noticeable impact on the quality of synthetic clinical letters: masking stopwords has a positive impact, while masking nouns or verbs has a negative effect; (5) The BERTScore should be the primary quantitative evaluation metric, with other metrics serving as supplementary references; (6) Contextual information has only a limited effect on the models' understanding, suggesting that synthetic letters can effectively substitute real ones in downstream NER tasks; (7) Although the model occasionally generates hallucinated content, it appears to have little effect on overall clinical performance. Unlike previous research, which primarily focuses on reconstructing original letters by training language models, this paper provides a foundational framework for generating diverse, de-identified clinical letters. It offers a direction for utilizing the model to process real-world clinical letters, thereby helping to expand datasets in the clinical domain. Our codes and trained models are available at https://github.com/HECTA-UoM/Synthetic4Health.

## 1 Introduction

With the development of medical information systems, electronic clinical letters are increasingly used in communication between healthcare departments. These clinical letters typically contain detailed information about patients’ visits, including their symptoms, medical history, medications, etc. (1). They also often include sensitive personal information, such as patients’ names, phone numbers, and addresses (2, 3). As a result, these letters are difficult to share and nearly impossible to use widely in clinical education and research.

In 2018, 325 severe breaches of protected health information were reported by CynergisTek (4) placing nearly 3,620,000 patients' records at risk (4). This data reflects just 1 year, and similar privacy breaches are unfortunately common. The most severe hacking incident affected up to 16,612,985 patients (4). Therefore, generating synthetic letters and applying de-identification techniques seem indispensable.

Additionally, due to privacy concerns and access controls, insufficient data remains a major challenge in clinical education, medical research, and healthcare system development (5). Some shared datasets offer de-identified annotated data, with the MIMIC series being a typical example. These datasets are accessible through PhysioNet. MIMIC-IV (6–8), the latest version, contains clinical data from 364,627 patients, collected from 2008 to 2019 at a medical center in Boston. It contains details about hospitalizations, demographics, and transfers. Numerous research studies have been conducted using this shared dataset. Another public dataset series in the clinical domain is i2b2/n2c2 (9), which is accessible through the DBMI Data Portal. This series includes unstructured clinical notes, such as process notes, radiology reports, and discharge summaries and is published for clinical informatics sharing and natural language processing (NLP) task challenges.

However, these shared datasets are often limited to specific regions and institutions, making them not comprehensive. Consequently, models and medical research outcomes derived from these datasets cannot be widely applied (10). Therefore, to address the lack of clinical datasets and reduce the workload for clinicians, it is essential to explore available technologies that can automatically generate de-identified clinical letters.

Existing systems generate clinical letters primarily by integrating structured data; however, there are not many studies that explore the use of natural language generation (NLG) models for this purpose (11–13). NLG attempts to combine clinical knowledge with general linguistic expressions to generate clinical letters that are both readable and medically accurate. However, NLG technology is not yet mature enough for widespread use in healthcare systems. Additionally, it faces numerous challenges, including medical accuracy, format normalization, and de-identification (12). Therefore, this investigation focuses on how NLG technology can be used to generate reliable and anonymous clinical letters, which can benefit medical research, clinical education, and clinical decision-making.

**Figure 1:**
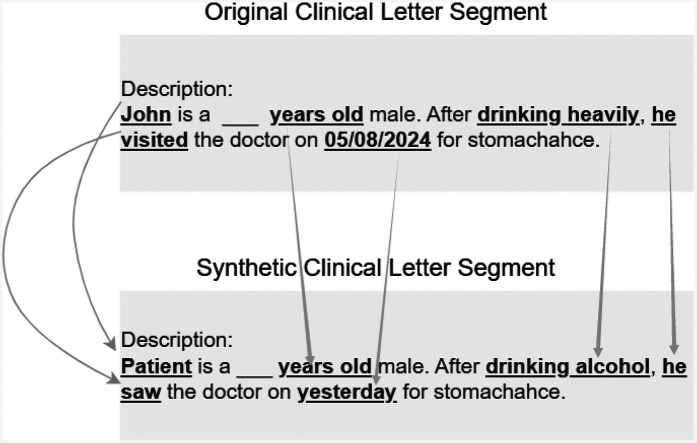
An example of the objective: sentence/segment-level generations.

The main aim of our work is to *generate de-identified clinical letters* that can *preserve clinical information* while *differing from the original* letters. A brief example of our objective is shown in Figure 1. Based on this objective, different generation models are explored as a preliminary attempt. Then, the best models are selected and various techniques are tested to improve the quality of the synthetic letters. The synthetic letters are evaluated not only with quantitative and qualitative methods but also in downstream tasks, i.e., NER. We hope this work contributes to addressing the challenge of insufficient data in the clinical domain.

In summary, this work is centered on the research question (RQ): “How can we generate reliable and diverse clinical letters without including sensitive information?” Specifically, it answers the following related sub-questions (RQs)^1^:

1. How do different models perform in masking and generating clinical letters?
2. How should the text be segmented in clinical letter generation?
3. How do different masking strategies affect the quality of synthetic letters?
4. How can we evaluate the quality of synthetic letters?

To answer these questions, we explored various large language models (LLMs) for masking and generating clinical letters, ultimately focusing on one that performed well. The overall highlights of this work are summarized as follows:

1. Mask and generate clinical letters using different LLMs at the sentence level.
2. Explore methods to improve synthetic clinical letters’ readability and clinical soundness.
3. Initially evaluate synthetic letters using both qualitative and quantitative methods.
4. Apply synthetic letters in downstream tasks and further evaluate them using clinically focused methods.
5. Explore post-processing methods to further enhance the quality of de-identified letters.

## 2 Background and literature review

We first introduce general language models, followed by their applications, especially within the clinical domain. We then present the generative language models based on the transformer architecture. These models serve as the technical foundation for most modern text generation tasks. Afterward, we review related works, discussing their relevance and connections to our work. Finally, all quantitative evaluation metrics used in this paper are introduced.

### 2.1 Development of language models (LMs)

The development of language models can be divided into three stages: rule-based approach, supervised modeling, and unsupervised modeling (14).

#### 2.1.1 Rule-based approach

The rule-based approach, first used in the 1950s, marks the beginning of NLP (15). This approach relies on a set of predefined rules, which were written and maintained manually by specialists (16, 17). Although it can generate standardized text without being fed with extensive input data (17), it has numerous limitations. Initially, manually crafted rules are often ambiguous, and the dependencies between different rules increase the cost of maintenance (15). Second, these stylized models cannot perform well in understanding realistic oral English and ungrammatical text, such as clinical discharge records, although these texts are still readable to humans (15). Third, they are not objective enough, as they are affected by the editors of the rule library. Additionally, they are not flexible enough to deal with special cases. Therefore, the rule-based method is only suitable for analyzing and generating highly standardized texts like prescriptions (17).

#### 2.1.2 Supervised language models

To address the limitations of the rule-based approach, supervised learning has been applied to NLP. The invention of statistical machine translation (SMT) in 1990 marked the rise of supervised NLP (14). It learns the correspondence rules between different languages by analyzing the input of bilingual texts (parallel corpora) (18). Supervised NLP models are trained on annotated labels to learn rules automatically. The learned rules will be used in word prediction or text classification. Hidden Markov model (HMM) and conditional random field (CRF) are two typical applications of this stage (19). Both of them work by tagging features of the input texts. HMM generates data by statistically analyzing word frequencies (20, 21). CRF, however, searches globally and calculates joint probabilities to get an optimal solution (22, 23). Long short-term memory (LSTM) is another typical example of supervised language modeling (24). In text generation tasks, the input consists of a set of labeled data or word vector sequences. By minimizing the loss between the predicted word vector and the actual word vector, LSTM can capture the dependencies between words in long texts (25, 26).

Although supervised language models perform better than the rule-based approach, domain experts still need to annotate the training dataset (14). In addition, collecting data in some domains is difficult due to privacy issues (such as *medical* and *legal* domains). This became an ongoing challenge in applying the supervised language models to specific tasks.

#### 2.1.3 Unsupervised language models

To address the high cost and difficulty of obtaining labeled data, unsupervised neural networks are applied to the language modeling (27). The popularity of corpora such as Wikipedia and social media provides enough data for training unsupervised models (14). Word embedding is a significant technique in this stage (28). For example, Word2Vec represents words using vectors with hundreds of dimensions. The context can be captured by training word vectors in a sliding window. By adjusting hyperparameters to maximize the conditional probability of the target word, the model can learn semantic information accurately (29, 30) [e.g., “Beijing”-“China”+“America” => “Washington” (31)]. After training, each word usually has a fixed word vector regardless of the context in which it appears (known as static word embedding) (26).

Unlike Word2Vec, BERT and GPT use contextual word embeddings, meaning that their word vectors reflect the semantic information and are affected by the context (32). BERT focuses on contextual understanding (33) (e.g., in the sentence “The bank is full of lush willows,” the word “bank” refers to a riverside rather than a financial institution). In contrast, GPT models focus on text generation within a specific context (34, 35) (e.g., Prompt: “Do you know Big Ben?” Answer: “Yes, I know Big Ben. It is the nickname for the Great Bell of the Clock located in London.”). Although unsupervised language models have been able to train and understand text proficiently, they still face challenges in practical applications, such as difficulty handling ambiguity and high computing resource consumption. Therefore, language modeling still has a long way to go.

### 2.2 Language models applications in clinical domain

Based on the modeling methods mentioned above, a variety of language models have been developed. They play an important role in scientific research and daily life, especially in the field of healthcare. In this section, we discuss the *clinical language model* applications in detail from two aspects: NER and NLG.

#### 2.2.1 Named entity recognition

NER was originally designed for text analysis and recognition of named entities, such as dates, organizations, and proper nouns (36). In the clinical domain, NER is used to identify *clinical events* (e.g., symptoms, drugs, treatment plans, etc.) from unstructured documents, along with their *qualifiers* (e.g., chronic, acute, mild), classify them, and extract the relationship between entities (37, 38). Earlier, NER systems relied on rule-based and machine learning methods that required extensive manual feature engineering. In 2011, Collobert et al. (39) used word embeddings and neural networks in NER. Since then, research in NER has shifted to automatic feature extraction.

**spaCy**^2^ is an open-source NLP library used for tasks like POS tagging and text classification. Additionally, it offers a range of pre-trained NER models. ScispaCy,^3^ a fine-tuned extension of spaCy on medical science datasets, can recognize entities such as “DISEASE,” “CHEMICAL,” and “CELL,” which are essential for medical research. Although NER is useful in rapidly extracting clinical terms, several challenges remain, such as non-standardization (extensive use of abbreviated words in clinical texts), misspellings (due to manual input by medical staff), and ambiguity (often influenced by context, e.g., whether the word “back” refers to an adverb or an anatomical entity) (37). Existing research mitigates these problems using *entity linking* (mapping extracted clinical entities to medical repositories such as UMLS and SNOMED). More deep learning models and text analysis tools are being developed to solve these issues.

#### 2.2.2 De-identification

The unprocessed clinical text poses a risk of personal information leakage. Additionally, manual de-identification is not only error-prone but also costly. Therefore, research on de-identification is indispensable for the secondary use of clinical data. Typically, de-identification is based on NER models to identify protected health information (PHI). Then, PHI is processed by different strategies (such as synonym replacement, removal, or masking) (40, 41).

Similar to NER, early de-identification approaches relied heavily on rule-based systems, machine learning, or hybrid models. PhysioNet DeID, the VHA best-of-breed (BoB), and MITRE’s MIST are three typical examples (42). However, these algorithms require extensive handcrafted feature engineering. With the development of unsupervised learning, recurrent neural networks (RNNs) and transformers are widely used in de-identification tasks (43, 44).

Philter, a protected health information filter (45), is a pioneering system that combines rule-based approaches with state-of-the-art NLP models to identify and remove PHI. Although Philter outperforms many existing tools like PhysioNet and Scrubber, particularly in terms of recall and F2 score, it still requires large amounts of annotated data for training (45). Additionally, research has shown that while the impact of de-identification on downstream tasks is minimal, it cannot be completely ignored (46). Therefore, performing de-identification without mistakenly removing semantic information is still a challenge in this field.

#### 2.2.3 Natural language generation

Both label-to-text and text-to-text generation are components of NLG (47). NLG consists of six primary sub-tasks, covering most of the NLG process. NLG architectures can generally be divided into three categories (47):

• **Modular architectures:** This architecture consists of three modules: the text planner (responsible for determining the content for generation), the sentence planner (which aggregates the synthetic text), and the realizer (which generates grammatically correct sentences). These modules are closely related to the six sub-tasks, and each module operates independently.
• **Planning perspectives:** This architecture considers NLG as a planning problem. It generates tokens dynamically based on the objectives, with potential dependencies between different steps.
• **Integrated or global approaches:** Currently the dominant architecture for NLG, this approach relies on statistical learning and deep learning. Common generative models, such as transformers and conditional language models, are included in this architecture.

In the field of healthcare, NLG applications include document generation and question-answering. Document generation involves discharge letters, diagnostic reports for patients, decision-making suggestions for experts, and personalized patient profiles for administrators (48). Some systems have already been implemented in practice. For instance, PIGLIT generates explanations of clinical terminology for diabetes patients (49), while MAGIC can generate reports for intensive care unit (ICU) patients (50). Question answering is another application of NLG. Tools like chatbots can provide patients with answers to basic healthcare questions (51).

Nowadays, NLG in the clinical field focuses on the development and training of transformer-based LLMs; examples of this work can be seen in (11, 52). These models perform well in specific domains such as semantic query (53) and electronic health record (EHR) generation (54). However, very few systems can reliably produce concise, readable, and clinically sound reports across multiple sub-domains (48).

### 2.3 Generative language models

#### 2.3.1 Transformer and attention mechanism

Although RNNs and LSTM networks are effective at capturing semantic understanding, their recursive structure not only prevents parallel computation but also makes them prone to gradient vanishing (55). The introduction of the transformer architecture in 2017 addressed this issue by replacing the recurrent structure with a multi-head attention mechanism (56). Since then, most deep learning models have been based on the transformer framework. Transformer architecture is based on an encoder–decoder model (56). To understand this, we first need to overview auto-regressive models and the multi-head attention mechanism.

Auto-regressive models’ predictions for each auto-regressive model token depend on the previous output. Therefore, it can only access the preceding tokens and operate iteratively. When the input sequence is *X*, the auto-regressive model aims to train parameters θ to maximize the log-likelihood of the conditional probability P (Equation 1) (56)

(1) $$L(X)=\sum_{i}\log\;P(x_{i}|x_{i-k},\ldots ,x_{i-1};\Theta )$$

**Multi-head attention mechanism:** The attention mechanism was initially proposed by Cho et al. (57). It can not only focus on the element being processed but also capture the context dependence (56). The scaled dot-product attention is computed as shown in Equation 2. Multi-head attention consists of several single-head attention (scaled dot-product attention) layers (56). Each word in the input sequence is converted into a high-dimensional vector representing semantic information by word embedding. These vectors are then passed through linear transformation layers to generate vectors for queries (Q), keys (K), and values (V). For each word, Q, K, and V are inputs to this single-head attention layer. The importance score of this word is calculated, and V corresponding to this word is multiplied to get the output of this head (called attention). Finally, outputs from all layers are concatenated to form a larger vector, which is the input to a feed-forward neural network (also the output of the multi-head attention layer) (56)

(2) $$\text{Attention}(Q,K,V)=\text{softmax}\left(\frac{QK^{T}}{\sqrt{d_{k}}}\right)V$$

**Transformer and pre-training language models (PLMs)**: Transformer consists of an encoder and a decoder. The auto-regressive model is the basis of the decoder. When the input sequence is $X=(x_{1},\ldots ,x_{N})$ and the output sequence is $Y_{M}=(y_{1},\ldots ,y_{M})$, the model can learn a latent feature representation $Z=(z_{1},\ldots ,z_{N})$ from X to Y. The generation of each new element $Y_{M}$ relies on the generated sequence $Y_{M-1}=(y_{1},\ldots ,y_{M-1})$ and feature representation Z. Both the encoder and the decoder use the multi-head attention mechanism (55, 56).

Many modern models are based entirely or partially on the transformer. They compute general feature representations for the training set by unsupervised learning. This is the concept of PLMs. They can be fine-tuned to adapt to the specific tasks on particular datasets (34, 55).

#### 2.3.2 Encoder-only models

Since the transformer’s encoder architecture can effectively capture the semantic features, some models only use this part for training. They are applied in text understanding tasks, such as text classification and NER. Bidirectional encoder representations from transformers (BERT) (58) is a representative model among them.

Unlike the transformer decoder, which uses an auto-regressive model, BERT is trained based on the masked language model (MLM) (34). It masks the word in the input sequence and uses the bidirectional encoder to understand the context semantically, which will be used in predicting the masked word (58). It has already been pre-trained on a 16 GB corpus. To deploy it, we only need to replace the original fully connected layer with a new output layer and then fine-tune the parameters on the dataset for specific tasks (58). This approach consumes fewer computing resources and less time than training a model from scratch. In the clinical domain, Bio_ClinicalBERT (59) and medicalai/ClinicalBERT (60) are fine-tuned in the clinical dataset based on the BERT architecture. Initially, due to BERT's focus on semantic understanding, it was rarely used for text generation (61).

Robustly optimized BERT pretraining approach (RoBERTa) (62) improved some key hyperparameters based on BERT. Instead of BERT’s static mask, it uses a dynamic mask strategy, which helps it better adapt to multitasking. Additionally, it gained a stronger semantic understanding after training on five English datasets of 160 GB. However, it was trained with more epochs and larger batch sizes compared to BERT, indicating higher computational resource requirements and longer training time (63).

To better handle long sequences, the Longformer introduces a sparse attention mechanism to reduce computation (64). This allows each token to focus only on nearby tokens rather than the entire sequence. Unlike traditional models like BERT and RoBERTa, which can only process no more than 512 tokens, the Longformer can handle up to 4,096 tokens. It consistently performs better than RoBERTa in downstream tasks involving long documents (64). The Clinical-Longformer model (65) was fine-tuned for the clinical domain.

Supplementary Table S1 summarizes the encoder-only models used in our work and their corresponding fine-tuning datasets.

#### 2.3.3 Decoder-only models

In 2020, the performance of ChatGPT-3 (66) in question answering task caught researchers’ attention to decoder-only architectures. As mentioned earlier, the transformer decoder is an auto-regressive model. It can only refer to the synthesized words on the left side to generate the new word, without considering the context (which is called masked self-attention). This method made it more flexible in generating coherent text. Compared with BERT, the GPT series performed well in zero-sample and small-sample learning tasks by enlarging the size of the model. Even without fine-tuning, a simple prompt can help GPT generate a reasonable answer (67).

Unlike GPT, which improves models’ performance by increasing dataset size and the number of parameters without limitations, Meta AI published a series of Llama models. These models aim to maximize the use of limited resources - in other words, by extending training, they reduce the overall demand on computing resources. The latest Llama3 model requires only 8–70 billion parameters (68), significantly less than GPT-3’s 175 billion (67). Additionally, it outperforms GPT-3.5 Turbo in five-shot learning (69).

#### 2.3.4 Encoder–decoder models

T5 family (70) is a classic example of the encoder–decoder model. This architecture is particularly suitable for text generation tasks that require deep semantic understanding (71). T5 transforms all kinds of NLP tasks into a text-to-text format (72). Unlike BERT, which uses word-based masking and prediction, T5 processes text at the *fragment* level using “span corruption” to understand semantics (72). For the fill-in-the-blank task, instead of replacing the specific words with <mask> like BERT, T5 replaces the text fragments with an ordered set of <extra_id_n> to reassemble the long sequence text. T5 needs to pre-process the input text according to the task requirements. A directive prefix should be added as a prompt.

Some language models fine-tuned with T5 on specific datasets, such as SciFive (fine-tuned in some science literature) (73) and ClinicalT5 (fine-tuned in clinical dataset MIMIC-III notes) (74), have shown excellent performance in their respective fields. The T5 family models used in this paper and their corresponding fine-tuned datasets are summarized in Supplementary Table S2.

#### 2.3.5 Comparison and limitations

According to Cai et al. (71), the encoder–decoder architecture performs best with sufficient training data. However, challenges in data collection can negatively affect its performance. Despite these challenges, different architectures are well-suited to different tasks. For example, for tasks requiring semantic understanding, such as text summarization, the encoder–decoder architecture is the most effective. In contrast, for tasks that involve minor word modifications, the encoder-only structure works better. However, the decoder-only structure is not suitable for tasks with insufficient training data and long text processing, but performs well in few-shot question answering tasks (71, 75).

Following these discussions, transformer-based PLMs have demonstrated strong performance in NLP tasks, but many challenges still remain.

### 2.4 Related works on clinical text generation

#### 2.4.1 LT3: label to text generation

LT3 (76) adopts an encoder–decoder architecture to generate synthetic text from labels. As shown in Supplementary Figure S1, labels such as medications are the input of the encoder, which can generate corresponding feature representations. The decoder generates prescription sequences based on these features. The pre-trained BERT tokeniser is used to split the input sequence into sub-words. LT3 is trained from scratch. Instead of using traditional greedy decoding, which may miss the global optimum, the authors proposed beam search decoding with backtracking (B2SD). This approach broadens the search range through a backtracking mechanism, preserving possible candidates for the optimal solution. To reduce time complexity, they used a probability difference function to avoid searching for low-probability words. Additionally, the algorithm penalizes repeated sub-sequences and employs a logarithmic heuristic to guide the exploration of generation paths. The authors test LT3 on the 2018-n2c2 dataset and evaluate the results using both quantitative metrics and downstream tasks. It was demonstrated that this model outperforms T5 in label-to-text generation.^4^

#### 2.4.2 Seq2Seq generation for medical dataset augmentation

Amin-Nejad et al. (75) compared the performance of the Vanilla transformer and GPT-2 using the MIMIC-III dataset in seq2seq tasks. Specifically, they fed as input a series of structured patient information as conditions, as shown in Supplementary Figure S2, to generate discharge summaries. They demonstrated that the augmented data outperforms the original data in downstream tasks (e.g., readmission prediction). Furthermore, they proved that the Vanilla transformer performs better with large samples, while GPT-2 excels in few-shot scenarios. However, GPT-2 is not suitable for augmenting long texts. Additionally, they used Bio_ClinicalBERT for the downstream tasks and discovered that Bio_ClinicalBERT outperformed the baseline model (BERT) in almost all experiments. This suggests that Bio_ClinicalBERT can potentially replace BERT in the biomedical field. Interestingly, although the synthetic data have a low score on internal metrics (such as ROUGE and BLEU), the performance on downstream tasks is notably enhanced. This may be because augmenting text can effectively introduce noise into the original text, improving the model’s generalization to unseen data.

According to their findings, decoder-only models like GPT-2 are not suitable for processing long texts. Bio_ClinicalBERT is particularly effective for tasks in the clinical area, and the Clinical transformer is promising in augmenting medical data. This provides more possibilities for our task of generating synthetic clinical letters.

#### 2.4.3 Discharge summary generation using clinical guidelines and human evaluation framework

Unlike the traditional supervised learning of fine-tuning language models (which requires a large amount of annotated data), Ellershaw et al. (77) generated 53 discharge summaries using only a one-shot example and a clinical guideline. Their research consists of two aspects: generating discharge summaries and a manual evaluation framework.

As shown in Supplementary Figure S3, the authors used clinical notes from MIMIC-III as input and incorporated a one-shot summary along with clinical guidance as prompts to generate discharge summaries by GPT-4-turbo. Initially, five sample synthetic summaries were evaluated by a clinician. Based on the feedback, the clinical guidance was revised to adapt to the generation task. Through iterative optimization, the revised guidance, combined with the original one-shot sample, became the new prompt. Then, the authors generated 53 discharge summaries using this method and invited 11 clinicians to do a final manual quantitative evaluation. Clinicians were invited to evaluate the error rate at the section level (e.g., diagnoses, social context, etc.). It includes four dimensions:

• Minor omissions,
• Severe omissions,
• Unnecessary text, and
• Incorrect additional text.

Each discharge summary was evaluated by at least two clinicians, and the authors calculated agreement scores to evaluate the subjectivity during the human evaluation stage. Unfortunately, the inter-rater agreement was only 59.72%, raising concerns that the revised prompts based on such feedback might result in subjective synthetic summaries. Although this study partially addresses the issue of insufficient training data and provides reliable human quantitative evaluation methods, it is still not well-suited for our investigation. Specifically, it required 11 clinicians to evaluate 53 synthetic samples, demonstrating the considerable time and manpower required. Therefore, there is still a long way to go before this technique can be used for large-scale text generation tasks.

#### 2.4.4 Comparison of masked and causal language modeling for text generation

Micheletti et al. (78) compared masked language modeling (MLM, including BERT, RoBERTa, BiomedNLP-PubMedBERT) and causal language modeling (CLM, including T5, BART, SciFive-large-Pubmed_PMC) across various datasets for masking and text generation tasks. They used qualitative and quantitative evaluations, as well as downstream tasks, to assess the quality of the synthetic texts. Their workflow is shown in Supplementary Figure S4. Based on these evaluations, the study yielded the following results:

• MLM models are better suited for text masking and generation tasks than CLM.
• Introducing domain-specific knowledge does not consistently improve model performance.
• Downstream tasks can adapt to the introduced noise. Although some synthetic texts might not achieve highly quantitative evaluation scores, they can still perform well in downstream tasks. This matches the findings from Amin-Nejad et al. (75).
• A lower random masking ratio (i.e., masked tokens/total tokens) can generate higher-quality synthetic texts.

These very recent findings provide insightful inspiration to our investigation. Our work builds on their research, expanding on masking strategies and focusing on the clinical domain.

## 3 Methodologies and experimental design

Due to the sensitivity of clinical information, many clinical datasets are not accessible. As mentioned in Section 2, numerous studies use NLG techniques to generate clinical letters and evaluate the feasibility of replacing the original raw clinical letters with synthetic letters. Most existing research involves fine-tuning PLMs or training transformer-based models from scratch on their datasets through supervised learning. These studies explore different ways to learn mapping from the original raw text to synthetic text and work on generating synthetic data that are similar (or even identical) to the original ones. Our work, however, aims to find a method that can generate clinical letters that can *keep the original clinical story, while not exactly being the same as the original letters*. To achieve this objective, we employed various models and masking strategies to generate clinical letters. The experiment follows these steps:

1. **Data collection and pre-processing**: We first accessed clinical letter examples (6–8) for an overview. The texts were segmented at the sentence level, and clinical entities and structural templates were extracted to capture the clinical narratives while maintaining clinical soundness.
2. **Randomly masking**: We randomly masked the context and generated clinical letters by predicting masked tokens using different LLMs.
3. **Model evaluation**: We evaluated synthetic letters generated by different language models. Based on their performance, we selected Bio_ClinicalBERT and worked on it.
4. **Masking strategy exploration**: We explored multiple masking strategies to retain clinical stories and diversity while removing private information. After generating clinical letters using these strategies, we evaluated their quality.
5. **Post-processing**: We applied post-processing techniques to further enhance the readability of synthetic letters.
6. **Downstream task evaluation**: We compared the performance of synthetic and original letters in a downstream NER task to evaluate the usability of these synthetic letters.

**Figure 2:**
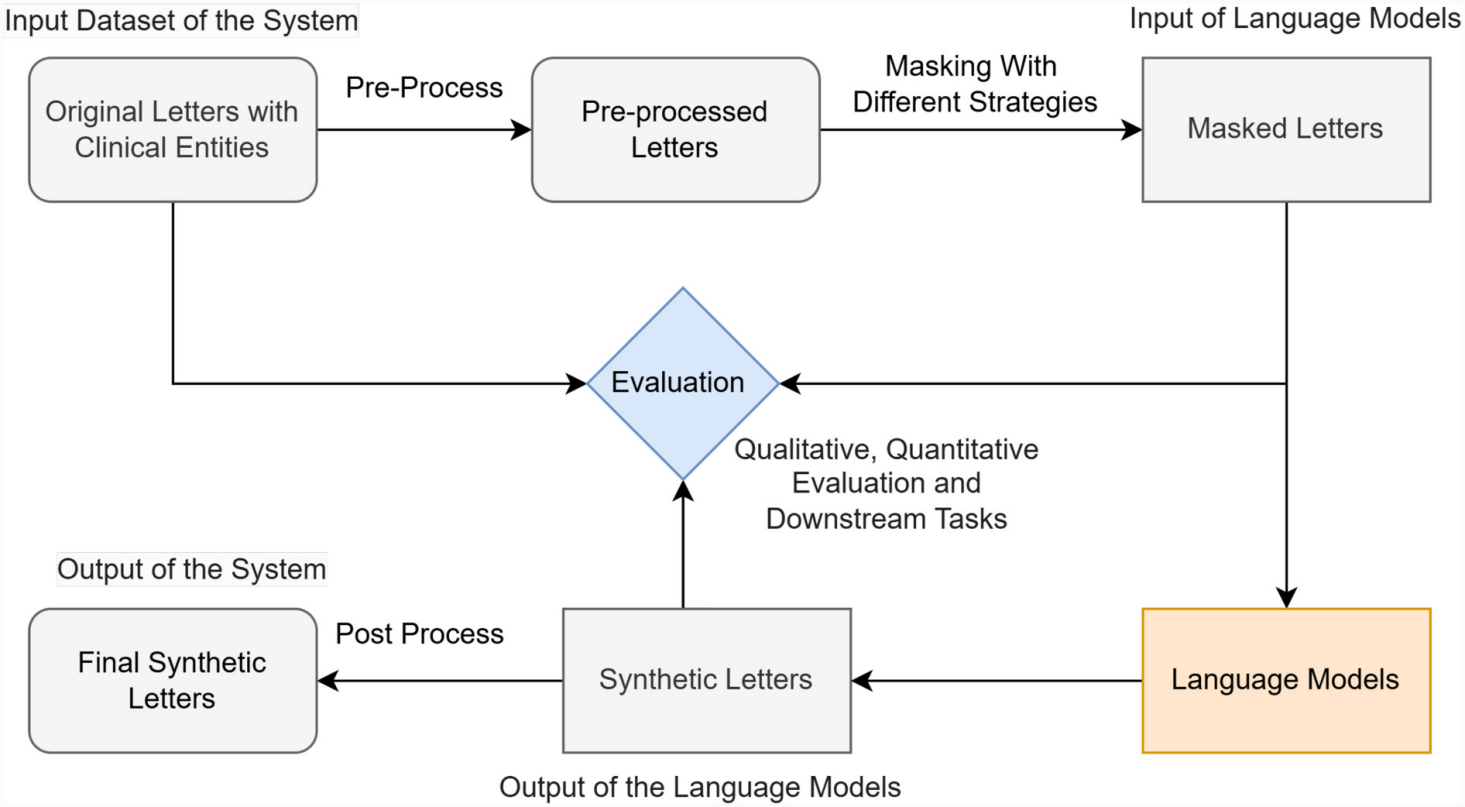
Overall investigation workflow for Synthetic4Health.

An overall investigation workflow is shown in Figure 2.

### 3.1 Dataset

Based on the objective of this project, we need a dataset that includes both clinical notes and some clinical entities. The dataset we used was from the SNOMED CT Entity Linking Challenge (6–8). It includes 204 clinical letters and 51,574 manually annotated clinical entities.

**Clinical letters:** The clinical letters were from a subset of discharge summaries in MIMIC-IV-Note (6, 79). It uses clinical notes obtained from a healthcare system in the United States. These notes were de-identified by a hybrid method involving the rule-based approach and neural networks. To avoid releasing sensitive data, the organization also did a manual review of PHI. In these letters, all PHI was replaced with three underscores “___.” The letters record the patient’s hospitalisation information (including the reason for visiting, consultation process, allergy history, discharge instructions, etc.). They are saved in a comma-separated value (CSV) format file “mimic-iv_notes_training_set.csv.” Each row of data represents an individual clinical letter. It consists of two columns, where the “note_id” column is a unique identifier for each patient’s clinical letter, and the “text” column contains the contents of the clinical letter. Since most language models have a limitation on the number of tokens to process (80), we tokenized the clinical letters into words using the “NLTK” library and found that all clinical letters contained thousands of tokens. Therefore, it is necessary to split each clinical letter into multiple chunks to process them. These separated chunks must be merged in the end to generate the whole letter.

**Annotated clinical entities:** The entities were manually annotated based on SNOMED CT. A total of 51,574 annotations cover 5,336 clinical concepts. They were saved in another CSV document which includes four columns: “note_id,” “start,” “end,” and “concept_id.” The “note_id” column corresponds to the “note_id” in the “mimic-iv_notes_training_set.csv” file. The “start” and “end” columns indicate the position of annotated entities. The “concept_id” can be used for entity linking with SNOMED CT. For example, for the “note_id” “10807423-DS-19,” the annotated entity “No Known Allergies” has a corresponding “concept_id”: “609328004.” This can be linked to SNOMED CT under the concept of “Allergic disposition” (81).

An example of text excerpted from the original letter is shown in Supplementary Figure S5. It contains the document structure and some free text. According to the dataset, document structure often corresponds to capital letters and colons “:.” Our primary goal is to mask the context that is neither part of the document structure nor annotated entities, and then generate a new letter, as both structure and clinical entities are essential for understanding clinical information (46).

### 3.2 Software and environment

All codes and experiments in this paper were carried out in the integrated development environment (IDE) “Google Colab Pro+” using a 52 GB system RAM and 225 GB disk space. The built-in T4 GPU (16 GB VRAM) accelerates the inference process. The primary tools used in the paper include:

• **Programming language and environment:** Python 3.10 serves as the main programming language.
• **Deep learning framework:** PyTorch 2.3.1 is the core framework used for loading and applying pre-trained language models (PLMs).
• **Natural language pocessing libraries:** This includes Hugging Face Transformers 4.42.4, NLTK (version ≥ 3.1), and BERTScore 0.3.13, among others. These are popular tools for text processing and evaluation in the NLP domain.
• **Auxiliary tools:** Libraries such as pandas (version ≥ 1.0.1) and mpmath (1.1.0 ≤ version < 1.4) can support data management, mathematical operations, and other routine tasks.

### 3.3 Pre-processing

**Figure 3:**
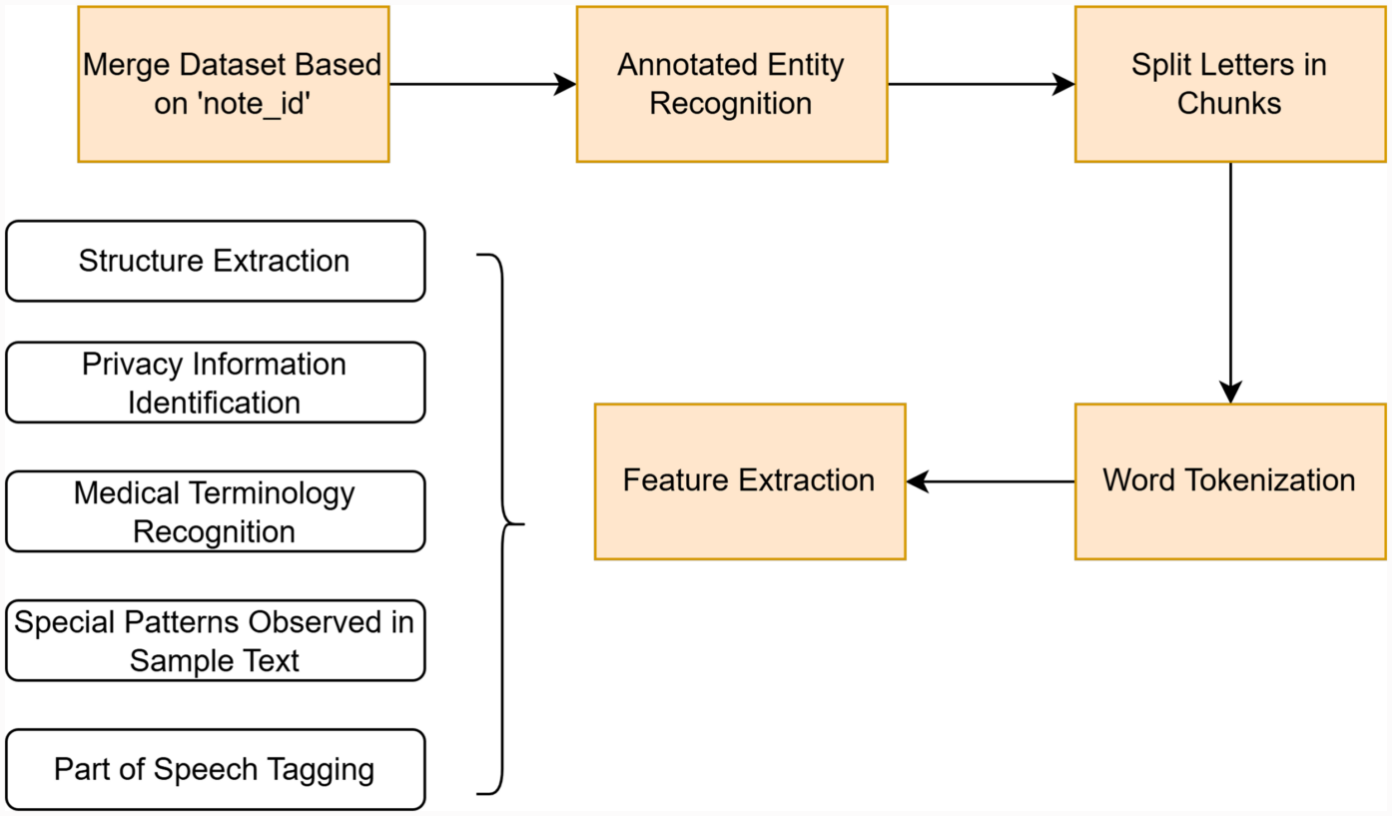
Pre-processing pipeline.

The collected dataset involves different files and comprises entirely raw data. It is necessary to pre-process these files before using them in generation tasks. The pre-processing of this system contains five steps: “Merge dataset based on ‘note_id,’” “Annotated Entity Recognition,” “Split Letters in Chunks,” “Word Tokenization,” and “Feature Extraction.” The pre-processing pipeline is shown in Figure 3.

#### 3.3.1 Merging dataset and annotated entity recognition

Initially, we merged the clinical letters file and annotations file into a new DataFrame. After this, we extracted manually annotated entities based on their index. An excerpt from an original letter is shown in Supplementary Figure S6, and the manually annotated entities are listed in Supplementary Table S3.

#### 3.3.2 Splitting letters into variable-length chunks

Typically, PLMs such as BERT, RoBERTa, and T5 have a limit on the number of input tokens, usually capped at 512 (82). When dealing with text that exceeds this limit, common approaches include discarding the excess tokens or splitting the text into fixed-length chunks of 512 tokens. In addition, some studies evaluate the tokens’ importance to decide which parts should be discarded (83).

In this work, each clinical letter (“note_id”) contains thousands of tokens, as mentioned in Section 3.1, to preserve as much critical clinical information as possible; therefore, we avoided simply discarding tokens. Instead, we adopted a splitting strategy based on semantics. Each block is not a fixed length. Rather, they are complete paragraphs that are as close as possible to the token limit. This approach aims to help the model better capture the meaning and structure of clinical letters, thereby improving its ability to retain essential clinical information while efficiently processing the text. In fact, we initially generated letters at the sentence level. However, it was found that processing at the sentence level is not only time-consuming but also fails to provide the model with enough information for inference and prediction. This is why the letters were processed in chunks rather than in sentences.

**Figure 4:**
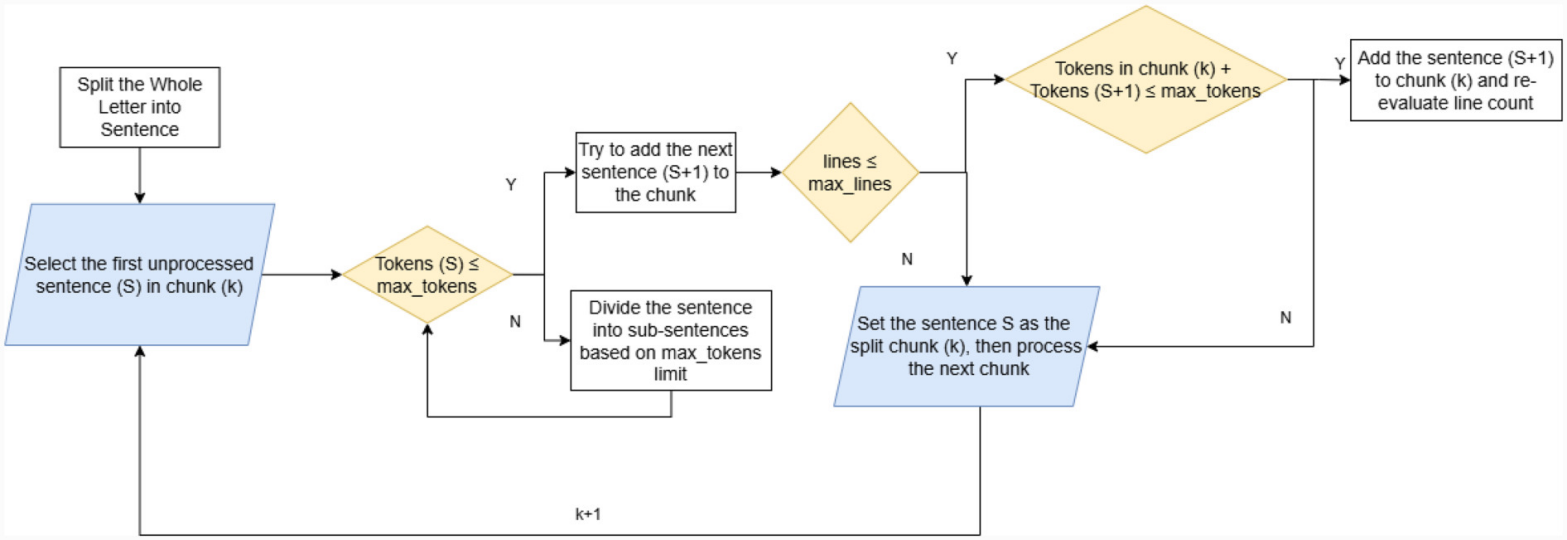
Text chunking workflow.

As shown in Figure 4, each raw letter is split into sentences first. We used the pre-trained models provided by the “NLTK” library, which combines statistical and machine-learning approaches to identify sentence boundaries. Each clinical letter is treated as a separate processing unit, with the first sentence automatically assigned to the first text block (chunk). To control the length of each chunk, we set a maximum line count parameter (max_lines). If the first sentence already meets the value of “max_lines,” the chunk will contain that single sentence only. Otherwise, subsequent sentences will be added to the chunk until the line count reaches the max_lines.

Extra care is needed when handling text with specific formats, such as medication dosage descriptions, as shown in Supplementary Figure S7. Because there is no clear sentence boundary, these sentences may exceed the tokens limitation. To address this, we first checked whether the sentence being processed exceeds the token limit (max_tokens). If it does not, the sentence will be added to the current chunk. Otherwise, the sentence should be split into smaller chunks, each no longer than “max_tokens.” This operation helps balance processing efficiency while maintaining semantic integrity. In the example shown in Supplementary Figure S7, although using line breaks to split the text seems to be more flexible, considering time complexity and the requirement to index the annotated entities, this method was not chosen.

#### 3.3.3 Word tokenization

To prepare the text for model processing, we split each chunk of text into smaller units: tokens. The tokenization methods can be categorised into two types: one for feature extraction and the other for masking and generation.

For the tokenization aimed at feature extraction, we used the “word_tokenise” method from the “NLTK” library. It is helpful to preserve the original features of the words, which is especially important for retaining clinical entities. For instance, in the sentence “Patient is a ___ yo male previously healthy presenting w/fall from 6 ft, from ladder.” Word boundaries such as spaces can be automatically detected for tokenization. The results of different tokenization methods are shown in the Supplementary Table S4.

As for the tokenization used for masking and generating, we retained the original models’ tokenization methods. The specific tokenization approach varies by model, as shown in Supplementary Table S4. For example, BERT family models use word-piece tokenization, which initially splits text by spaces and then further divides the words into sub-words (62). This approach is particularly effective for handling words that are not in the pre-training vocabulary and is especially useful for predicting masked words. For complex clinical terms, however, these models rely heavily on a predefined dictionary, which can result in unsatisfactory tokenization and hinder the model’s understanding. For instance, the word “COVID-19” is tokenized by BERT into [“co,” “##vid,” “–,” “19”]. In contrast, the T5 family models use sentence-piece tokenization. It does not rely on space to split the text. Instead, this method tokenises directly from the raw text, making it better suited for handling abbreviations and non-standard characters (e.g., “COVID-19”), which are common in clinical letters.

It is important to note that although all BERT family models use word-piece tokenization, the results can still differ. This is because different models use different vocabularies during pre-training, leading to variations in tokenization granularity. The tokenization methods for each model are detailed in Supplementary Table S4. Each tokenization approach has its own advantages and disadvantages for processing clinical letters. Therefore, exploring how these models impact the clinical letter generation is also a requirement of our project.

#### 3.3.4 Feature extraction

Since we aimed to generate de-identified clinical letters that can preserve clinical narratives during masking and generation, it is necessary to extract certain features beforehand. We extracted the following features, with an example provided in Supplementary Figure S8 and Supplementary Table S5.

• **Document structure:** This feature is identified by a rule-based approach. As mentioned in Section 3.1, structural elements (or templates) often correspond to the use of colons “:.” They should not be masked to preserve the clinical context.
• **Privacy information identification:** In this part, we used a hybrid approach. To identify sensitive information such as “Name,” “Date,” and “Location (LOC),” we employed a NER toolkit from Stanza (84). To handle privacy information like phone numbers, postal codes, and e-mail addresses, we implemented a rule-based approach. Specifically, we devised several regular expressions to match the common formats of these data types. These pieces of private information should be masked.
• **Medical terminology recognition:** A NER toolkit pre-trained on the i2b2 dataset is used here (85). It can identify terms like “Test,” “Treatment,” and “Problem” in free text. Although our dataset has already been manually annotated, these identified terms can serve as a supplement to the pre-annotated terms.
• **Special patterns observed in sample text:** Some specific patterns, like medication dosages (e.g., enoxaparin 40 mg/0.4 ml) or special notations (e.g., “b.i.d.”), may carry significant meaning. We retained these terms unless they were identified as private information to preserve the clinical background of the raw letters.
• **Part of speech (POS) tagging:** Different parts of speech (POS) play distinct roles in interpreting clinical texts. We aimed to explore how these POS influence the model’s understanding of clinical text. To achieve this, we used a toolkit (85) trained on the MIMIC-III (86) dataset for POS tagging. It performs better than SpaCy^5^ and NLTK in handling clinical letters.

### 3.4 Clinical letter generation

We discuss the models and masking strategies that are used in generating synthetic clinical letters. It is important to clarify that our key objective is to generate letters that differ from the original ones, rather than being exact copies, as the same statement may indirectly reveal the patients’ privacy. Although fine-tuning the model can always improve precision and enhance the model’s semantic comprehension ability, it tends to produce letters that are too closely aligned with the originals. This also causes the fine-tuned model to rely too heavily on the original dataset, compromising its ability to generalize. Therefore, simply fine-tuning the model is not ideal if the PLMs can already generate the readable text. Instead, we should concentrate on how to *protect clinical terms and patient narratives as well as avoid privacy breaches*.

As discussed in Sections 2.3 and 2.4, decoder-only models struggle with processing long texts that require contextual understanding (75). Additionally, deploying them requires substantial computing resources and time. Therefore, we explored various PLMs, including both encoder-only and encoder–decoder models, in this paper. After evaluating their ability to generate synthetic letters from our dataset, we focused on Bio_ClinicalBERT, a well-performed model in our task, to experiment with different masking strategies. Additionally, from the discussion in Section 3.3, we need to split the text into various-length-chunks. So, the appropriate *length of these chunks* is also experimented with Bio_ClinicalBERT.

#### 3.4.1 Encoder-only models with random masking

As mentioned earlier, the primary method for this paper involves masking and generation. We focused extensively on encoder-only models because of their advantage in bi-directional semantic comprehension. These encoder-only models, including BERT, RoBERTa, and Longformer (detailed in Section 2.3) were compared for their performance. Given the clinical focus of this task, we particularly explored model variants that were fine-tuned on clinical or biological datasets. However, as no clinically fine-tuned RoBERTa (62) variant was available, the RoBERTa-base was used for comparisons. Specifically, the encoder-only models we explored include Bio_ClinicalBERT (59), medicalai/ClinicalBERT (60), RoBERTa-base (62), and Clinical-Longformer (65).

We used the standard procedure for masked language modeling (MLM). First, the tokens that need to be masked were selected. They were then corrupted, resulting in masked text that includes both masked and unmasked tokens. Next, the model predicts the masked tokens and replaces them with the ones having the highest probabilities.

#### 3.4.2 Encoder–decoder models with random masking

Although encoder–decoder models are not typically used for masked language modeling, they are well-suited for text generation. The architecture of T5, in particular, is designed to maintain the coherence of the text (70). Therefore, we included the T5 family models for comparisons.

**Figure 5:**
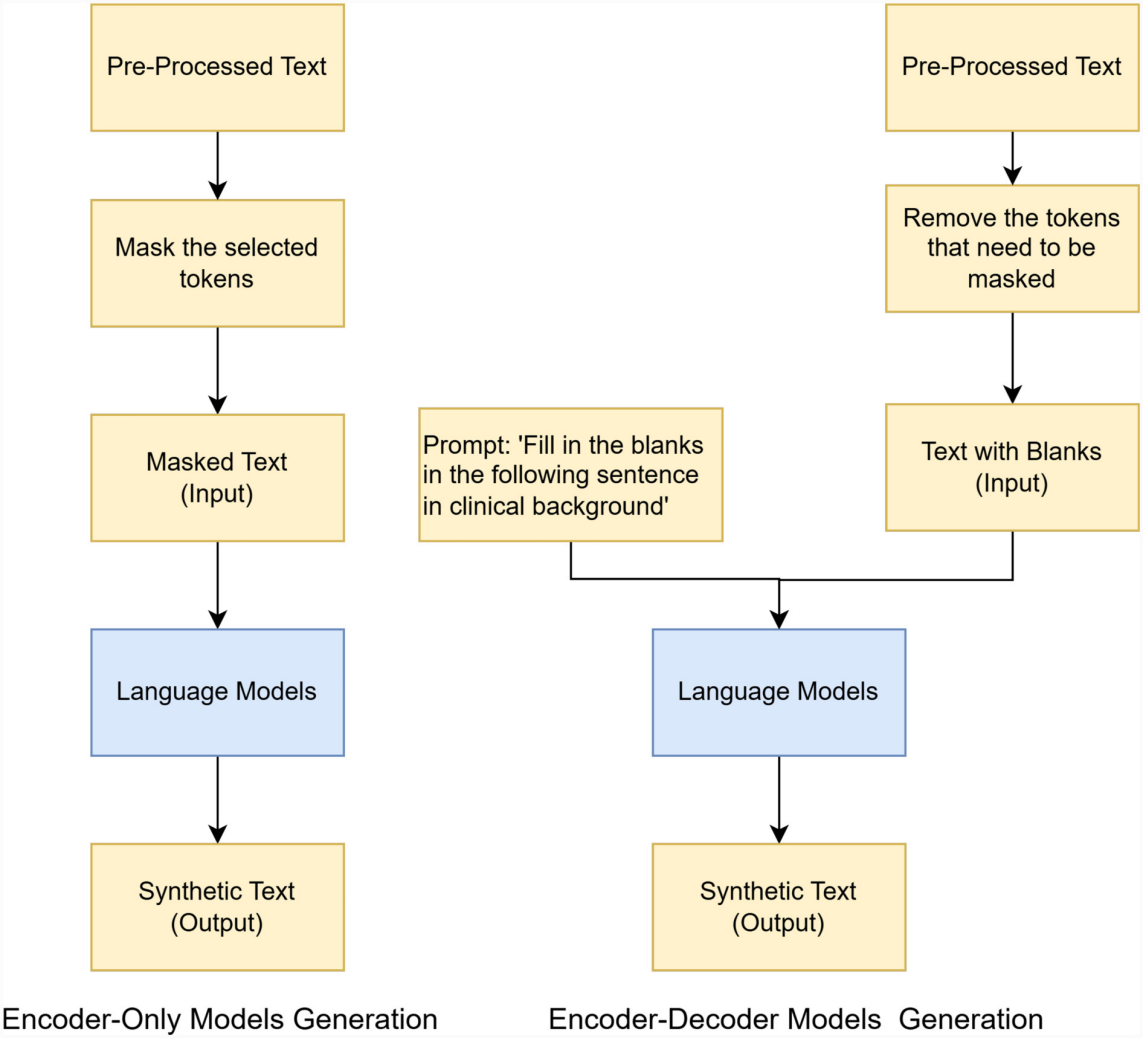
Comparison of encoder-only and encoder–decoder model architectures.

The process of generating synthetic letters with encoder–decoder models is very similar to that with encoder-only models. The difference is that, unlike the BERT family, which automatically masks tokens and replaces them with “<mask>,” the T5 family models *do not have any built-in masking function*. As a result, we identified the words that needed to be masked by index and removed them, which are represented as “extra_id_x” in the T5 family models. The text, with these words removed, was then used for generation, which we refer to as “text with blanks.” To maintain consistency in the format, we later replaced “extra_id_x” with “<mask>” when displaying the masked text. Additionally, the T5 family models require a prompt as part of the input. For this task, the complete input was structured as “Fill in the blanks in the following sentence in the clinical background” + “text with blanks.” In this paper, we used T5-base (70), Clinical-T5-Base (87, 88), Clinical-T5-Sci (87, 88), and Clinical-T5-Scratch (87, 88) for comparison. The comparison of encoder-only and encoder–decoder model architectures is shown in Figure 5.

#### 3.4.3 Different masking strategies with Bio_ClinicalBERT

To make the synthetic letters more readable, clinically sound, and privacy-protective, different masking strategies were tested based on the following principles.

1. **Preserve annotated entities:** The manually annotated entities should not be masked to retain the clinical knowledge and context.
2. **Preserve extracted structures:** Tokens that are part of the document structure should be preserved as templates for clinical letters.
3. **Mask detected private information:** This is helpful in de-identification. Although the dataset we use is de-identified, this approach may be useful when this system is deployed with real-world data.
4. **Preserve medical terminology:** It still aims to retain clinical knowledge, as some diseases and treatments were not manually annotated.
5. **Preserve non-private numbers:** Certain numbers, such as drug dosage or heart rates, are indispensable for clinical diagnosis and treatment. However, only non-private numbers should be retained, while private information (such as phone numbers, ages, postal codes, dates, and email addresses) should be masked.
6. **Preserve punctuation:** Punctuation marks such as periods (“.”) and underscores (“___”) should not be masked, as they clarify the sentence boundaries and make the synthetic letters more coherent (89).
7. **Retain special patterns in samples:** Tokens that match specific patterns (e.g., “Vitamin C ˆ1,000 mg,” “Ibuprofen > 200 mg,” etc.) should be retained, as they may contain important clinical details. These patterns are summarized by analyzing raw sample letters.

Based on the principles above, different masking strategies were experimented with:

1. **Mask randomly:** Tokens that can be masked are selected randomly from the text. We experimented with *masking ratios* ranging from 0% to 100% in 10% increments. This approach helps to understand how the number of masked tokens influences the quality of synthetic letters and provides a baseline for other masking strategies.
2. **Mask based on POS tagging:** We experimented with different configurations in this section, such as masking only nouns, only verbs, etc. It is helpful to understand how POS influences the models’ context understanding. Similar to the random masking approach, we selected the tokens based on their POS configuration and masked them in 10% increments from 0% to 100%.
3. **Mask stopwords:** Stopwords generally contribute little to the text’s main idea. Masking stopwords serves two purposes: reducing the *noise* for model understanding and increasing the *variety* of synthetic text by predicting these words. Moreover, they do not influence crucial clinical information. This approach is highly similar to the one used in “Mask based on POS tagging.” The only difference is the criteria for selecting tokens. Specifically, tokens are selected based on whether they are stopwords rather than on their POS. The “NLTK” library was used for detecting stopwords in the text.
4. **Hybrid masking using different ratio settings:** After employing the aforementioned masking strategies, we observed the influence of these elements. Additionally, we experimented with their *combinations* at different masking ratios based on the outcomes, such as masking 50% nouns and 50% stopwords simultaneously.

#### 3.4.4 Determining variable-length chunk size with Bio_ClinicalBERT

As mentioned in Section 3.3, we utilize two parameters in our chunk segment procedure: “max_lines” and “max_tokens.” “max_lines” represents the desired length of each chunk, while “max_tokens” is related to the computing resources and model limitations. These two parameters determine the final length of each chunk together. Although most models we used have a limit of 512 tokens (except for the Longformer, which can process up to 4,096 tokens), we set 256 as the value for “max_tokens” due to computing resource constraints.

As for “max_lines,” we experimented with values starting from 10 lines, increasing by 10 lines each time, and calculated the average tokens for each chunk. Once the token growth began to slow, we refined the search by using finer increments. Finally, we selected the number of lines at which the average tokens per chunk stopped growing. This is because more lines in each chunk provide more information for the model to predict masked tokens. However, if the chunk length reaches a critical threshold, it indicates that the primary limitation is “max_tokens” not “max_lines.” Continuing to increase “max_lines” would lead to additional computational overhead, as the system would have to repeatedly check whether adding the next sentence meets the required line count.

### 3.5 Evaluation methods

**Figure 6:**
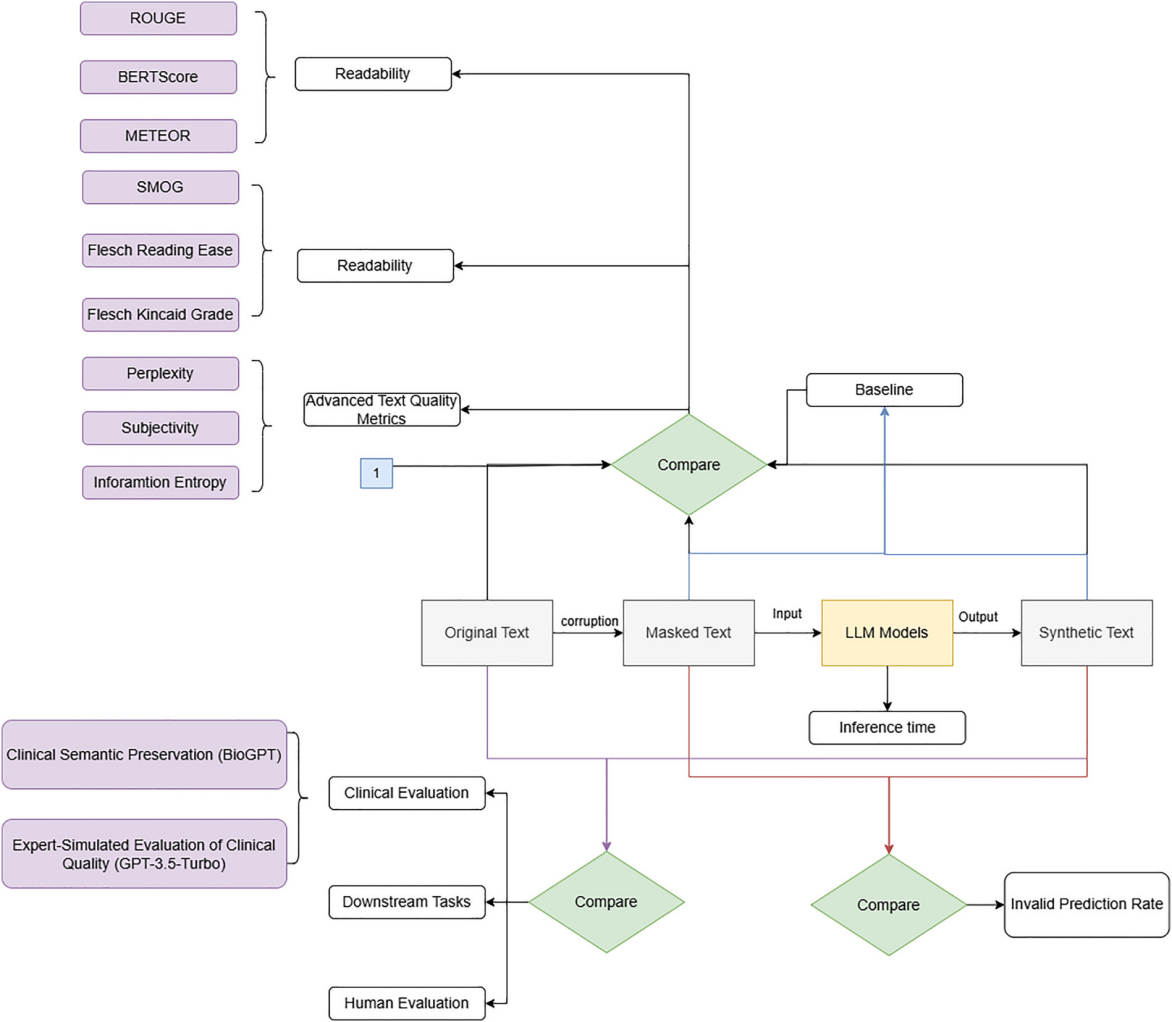
Evaluation pipeline.

Both quantitative and qualitative methods will be used to evaluate the performance. Additionally, a downstream task (NER) is employed to assess whether the synthetic clinical letters can replace the original raw data. The evaluation methods pipeline is illustrated in Figure 6.

#### 3.5.1 Quantitative evaluation

To comprehensively evaluate the quality of the synthetic letters, we used quantitative evaluation from multiple dimensions, including the model’s inference performance, the readability of the synthetic letters, and their similarity to the raw data. The specific metrics are listed in the following.

**Standard NLG metrics:** It covers standard NLG evaluation methods such as ROUGE, BERTScore, and METEOR. ROUGE measures literal similarity, the BERTScore evaluates semantic similarity, and METEOR builds on ROUGE by taking synonyms and word order into account. It provides a more comprehensive evaluation of the synthetic text (90).

These evaluations are performed by comparing the synthetic text with the original text. Moreover, a baseline is calculated by comparing the masked text to the original text. The evaluation score should exceed the baseline but remain below “1,” ensuring that it does not exactly replicate the original text.

**Readability metrics:** To evaluate the readability, we calculated SMOG, Flesch Reading Ease, and Flesch–Kincaid Grade Level. Given our clinical focus, we prioritized SMOG as the primary readability metric, with Flesch Reading Ease and Flesch–Kincaid Grade Level as reference standards. In this analysis, we compared the readability metrics of the synthetic text with those of the original and masked texts. The evaluation results should closely approximate the original text’s metrics. Significant differences (91)^6^ may suggest that the model cannot preserve semantic coherence and readability adequately.

**Advanced text quality metrics:** In this part, we calculated the perplexity, subjectivity, and information entropy. We want the synthetic letters to be useful in training clinical models. Therefore, perplexity should not be far away from the value of the original letters. As for subjectivity and information entropy, we expect the synthetic letters to be both subjective and informative.

**Invalid prediction rate:** We calculated the invalid prediction rate for each generation configuration. This ratio is determined by dividing the number of invalid predictions (such as punctuation marks or subwords) by the total number of masked words that need to be predicted. We expect the model to generate more meaningful words. Since punctuation marks are not masked, the model should avoid generating too many non-words. This metric can provide insights into the model’s inference capability.

**Inference time:** The inference time for each generation configuration across the whole dataset (204 clinical letters) was recorded. Shorter inference times indicate lower computational resource consumption. When this system is deployed on large datasets, it is expected to save both time and computing resources.

#### 3.5.2 Qualitative evaluation

In the quantitative evaluation, we not only calculated the evaluation metrics for the entire dataset but also recorded the results for each individual synthetic clinical letter. Interestingly, while some synthetic texts exhibited strong performance according to most metrics, they did not always appear satisfactory upon “visual” inspection. Conversely, some synthetic letters with average metrics may appear more visually appealing.

Although human evaluation is the most reliable approach for evaluating clinical letters, it is limited by availability and cost. Therefore, combining qualitative and quantitative evaluations helps in identifying suitable quantitative metrics for assessing the performance of our model. Once identified, one of these metrics can be used as the primary standard, while the others serve as supporting indicators. As a workaround, we selected a small sample of representative clinical letters based on the evaluation results. Subsequently, we reviewed the outcomes to better understand how different generation methods impacted these results, while also evaluating their correspondence with the quantitative metrics.

#### 3.5.3 Downstream NER task

Beyond qualitative and quantitative evaluation, we can also apply synthetic clinical letters in a downstream NER task. This is helpful to further evaluate their quality and their potential to replace original ones in clinical research and model training.

ScispaCy^7^ and spaCy^8^ are used in this part. As shown in Supplementary Figure S9, they extract features from the text and learn the weights of each feature through neural networks. These weights are updated by comparing the loss between the predicted probabilities and actual labels. If a word does not belong to any label, it is classified as “O” (outside any entity). spaCy initializes these weights randomly. However, the version of ScispaCy we used, “en_ner_bc5cdr_md,” is specifically fine-tuned on the BC5CDR corpus. It focuses more on “chemical” and “disease” entities while retaining the original general features.

**Figure 7:**
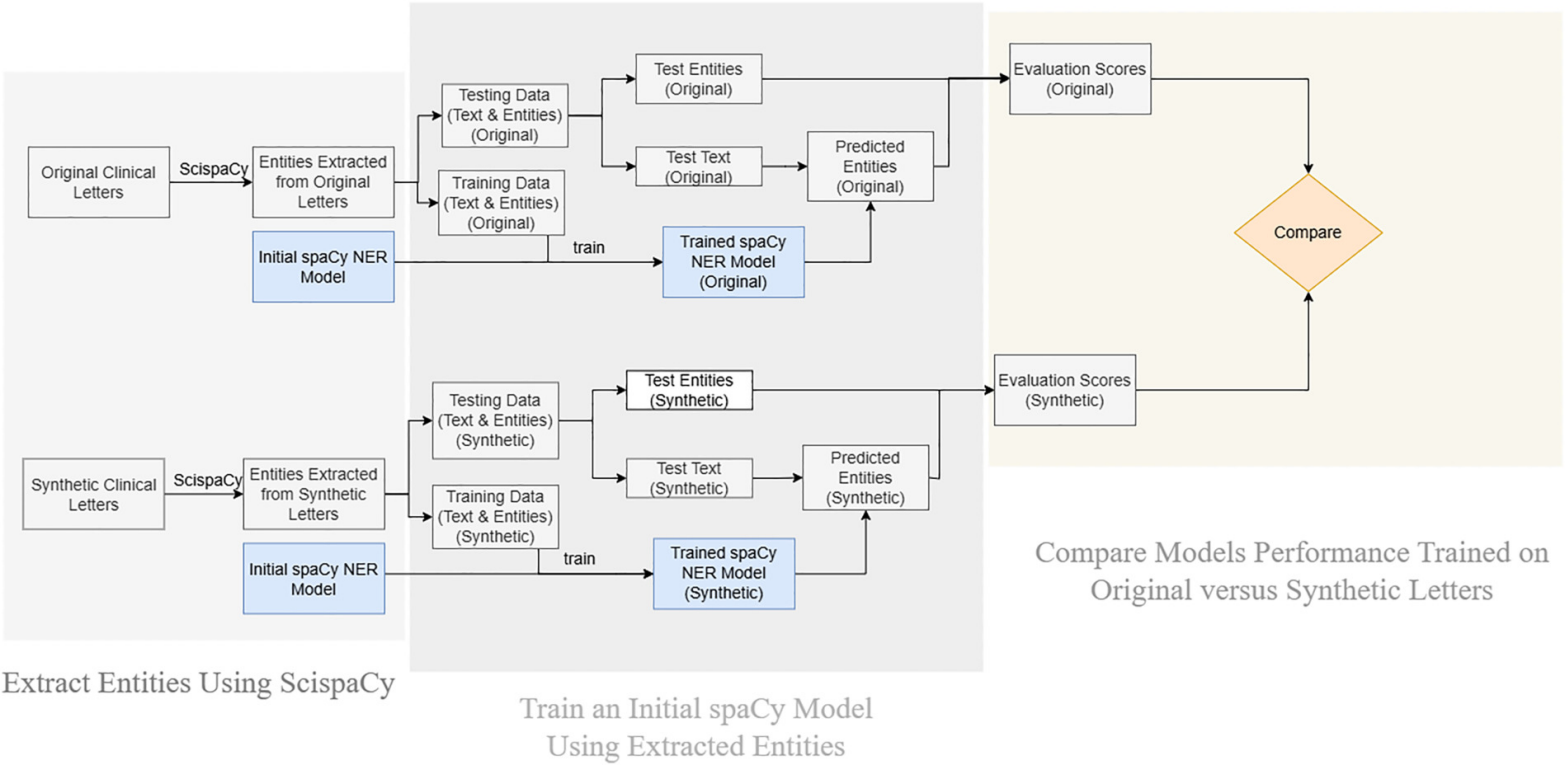
Workflow of the downstream NER task.

In this downstream NER task, as shown in Figure 7, we initially extracted entities from letters using ScispaCy. Subsequently, these entities were used to train a base spaCy model. The trained model was then employed to extract entities from the testing set. Finally, we compared these newly extracted entities with those originally extracted by ScispaCy, and the evaluation scores were calculated. These steps were performed on both original clinical letters and synthetic letters, to assess whether the synthetic letters can potentially replace the original ones.

#### 3.5.4 Clinical evaluation

**Clinical semantic preservation:** To evaluate how much clinical information is preserved, we used BioBERT (52) for a rough estimate. Specifically, we tokenized both the original and synthetic letters, obtained their embeddings using BioBERT, and computed the cosine similarity between them. Since BioBERT is trained on biomedical corpora, its embeddings are expected to capture clinical semantic features. A high similarity score indicates that clinical information is largely preserved. However, it is important to note that this method only evaluates the effectiveness of preserving clinical narratives at the semantic level and does not guarantee medical factuality.

**Figure 8:**
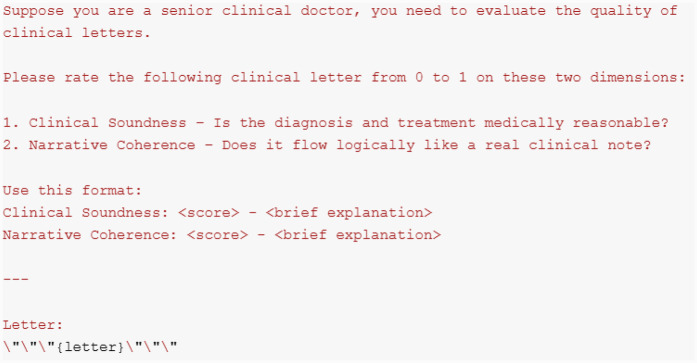
GPT-3.5-turbo prompt for clinical evaluation.

**Expert-simulated evaluation of clinical quality:** To further evaluate the clinical usefulness of our synthetic letters, we employed GPT-3.5-Turbo (92) through prompt-based evaluation. Specifically, we evaluated the results from two perspectives: clinical soundness and narrative coherence. Clinical soundness measures whether the content aligns with medical factuality, while narrative coherence evaluates whether the letter is contextually consistent and resembles a real-world clinical letter. The prompt we used is shown in Figure 8.

### 3.6 Post-processing

#### 3.6.1 Filling in the blanks

As described in Section 3, the dataset we used has been de-identified with all private information replaced by three underscores “___.” We hope that the synthetic clinical letters can maintain a certain degree of clinical integrity without disclosing any private patient information. To address this, a post-processing step was added to the synthetic results. This step involves masking the three underscores (“___”) detected and using PLMs to predict the masked part again. For example, if the original text is “___ caught a cold,” the post-processing result should ideally be “John caught a cold” or “patient caught a cold.” Such synthetic clinical letters can better support clinical model training and teaching.

In this part, we used Bio_ClinicalBERT and BERT-base models. Although Bio_ClinicalBERT is better at clinical information understanding, this issue is not directly related to clinical practice, so we used BERT-base for comparison.

#### 3.6.2 Spelling correction

Since our data come from real-world sources, it is inevitable that some words may be misspelled by doctors. These spelling errors can negatively impact the model’s training process or hinder clinical practitioners’ understanding of the synthetic clinical letters. Although some errors are masked and re-generated, our masking ratio is not always 100%, so some incorrect words may still exist. Toolkit “TextBlob” (93) was added to correct these errors. Specifically, it uses a rule-based approach that relies on a built-in vocabulary library to detect and correct misspellings.

### 3.7 Summary

In this section, we present the experimental design and subsequent implementation steps: these include defining project requirements, data collection and environmental setup, pre-processing, masking and generating the text, post-processing, the downstream NER task, clinical evaluation, and both qualitative and quantitative assessments. An example of the entire process flow is shown in Supplementary Figure S10.

## 4 Experimental results and analysis

### 4.1 Chunk segmentation effects on inference time

As mentioned in Section 3.4.4, we set “max_lines” as a variable and “max_tokens” equal to 256. A series of increasing “max_lines” were tested until the average tokens per chunk peaked. We initially did this on a small sample (seven letters). The results are shown in Supplementary Table S6 for the Bio_ClinicalBERT model.

We can see that the average tokens per chunk reaches a peak as the “max_lines” parameter increases to 41. Similarly, inference time decreases as “max_lines” increases to 41, but it increases again once it exceeds this value. This experiment was also conducted on slightly larger samples of 10 and 30 letters. All of them showed the same trend. However, the inference time here may only reflect an overall trend, not exact results, as it is influenced by many factors, not only the chunk size but also the internet speed.

### 4.2 Random masking: qualitative results

We employed both encoder-only and encoder–decoder models to mask and generate the data, yielding numerous interesting results for human evaluation. Given space constraints, only a simple example is provided here. Following the masking principles in Section 3.4, the eligible tokens were randomly selected for masking. Although the initial intention was to mask 50% of tokens, the actual masking ratio was lower due to the requirement to preserve certain entities and structures.

#### 4.2.1 Encoder-only models

**Figure 9:**
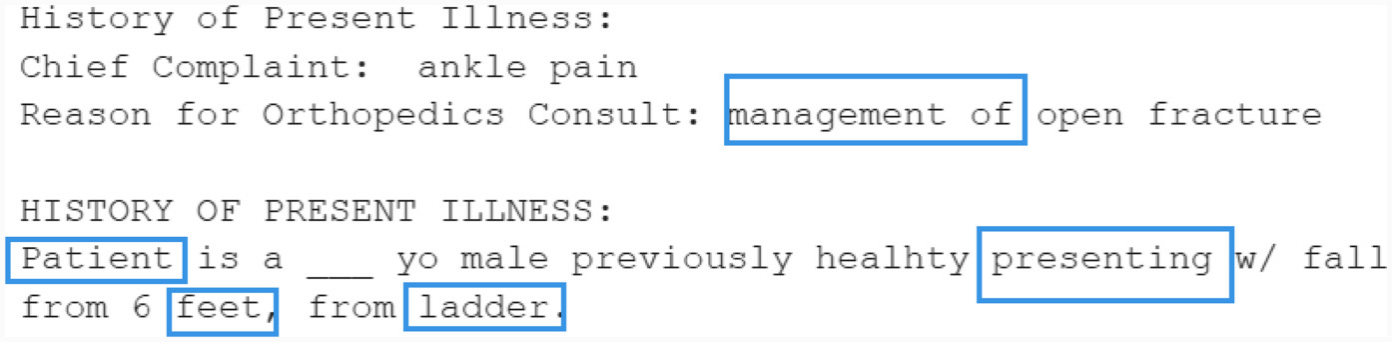
Original unprocessed example sentence (6–8) (“note_id”: “10807423-DS-19”) (the circled tokens will be masked).

**Figure 10:**
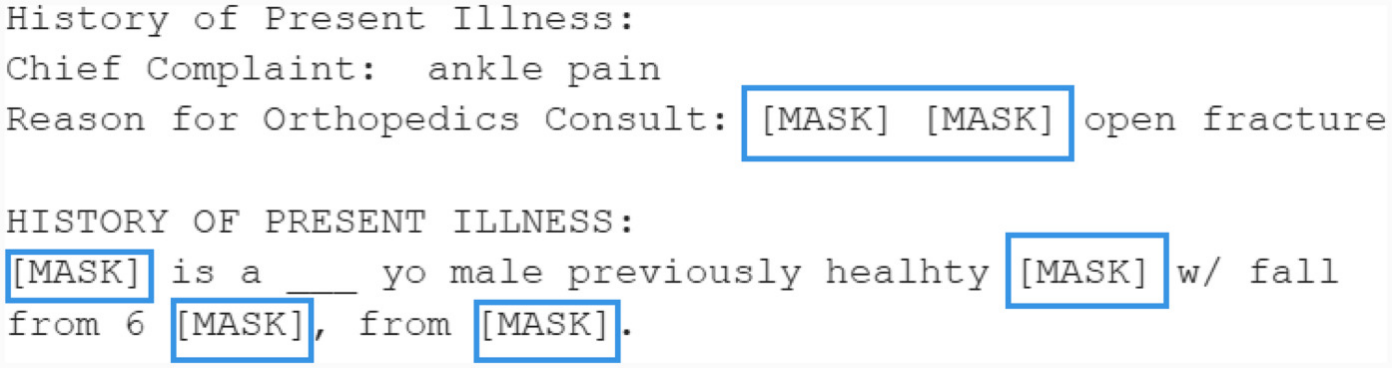
An example of the masked sentence.

The original sentence is displayed in Figure 9. After feature extraction, the resulting structure is shown in Supplementary Figure S11. As detailed in Supplementary Table S7, certain manually annotated entities are excluded from masking. The output of this masking process is shown in Figure 10.

**Figure 11:**
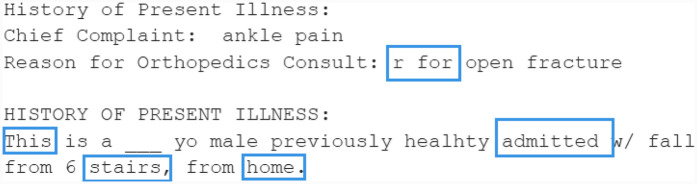
Example sentence generated by Bio_ClinicalBERT.

The generated text using Bio_ClinicalBERT is displayed in Figure 11. For “management of open fracture,” the model produced “r,” which is commonly used to denote “right” in clinical contexts, showing a relevant and logical prediction. Furthermore, the model’s input “R ankle,” despite not being in the figure due to space constraints, provided context for predicting “r” instead of “left.” Interestingly, the term “admitted” was generated, even though it was not in the input, indicating the model’s understanding of clinical context. Although the phrase “from 6 stairs, from home” is entirely different from the original (“from 6 feet, from ladder”), it remains contextually appropriate.

Overall, Bio_ClinicalBERT produced a clinically sound sentence, even though no tokens matched the original. In other examples, the predicted words may partially overlap with the original text. Nonetheless, this model effectively retains clinical information and introduces diversity without altering the text’s meaning.

The results from medicalai/ClinicalBERT and Clinical-Longformer are shown in Supplementary Figures S12 and S13. All three clinical-related models correctly predicted “r” from the input context. The medicalai/ClinicalBERT model performs *comparably* to Bio_ClinicalBERT, despite adding an extra comma, which did not affect the text’s clarity. However, Clinical-Longformer’s predictions, while understandable, were *repetitive* and less satisfactory. Importantly, none of these three models altered the original meaning.

The result generated by RoBERTa-base is shown in Supplementary Figure S14. While the generated text initially seems reasonable, the predicted word “years” shifts the focus to a temporal context, which was not intended. This is likely because RoBERTa is pre-trained on a general corpus and lacks sufficient clinical knowledge for accurate text generation, or it could simply be a coincidence based on this specific sentence, where RoBERTa-base inferred “years” from its training data.

#### 4.2.2 Decoder-only GPT-4o

Additionally, GPT-4o was used for comparison, with the prompt “Replace ‘<mask>’ with words in the following sentence:.” The results, shown in Supplementary Figure S15, are satisfactory. As discussed in Section 2.3, decoder-only models excel in few-shot learning (67), which is confirmed by this experiment. However, its performance may decline with long clinical letters (75).

#### 4.2.3 Encoder–decoder models

**Figure 12:**
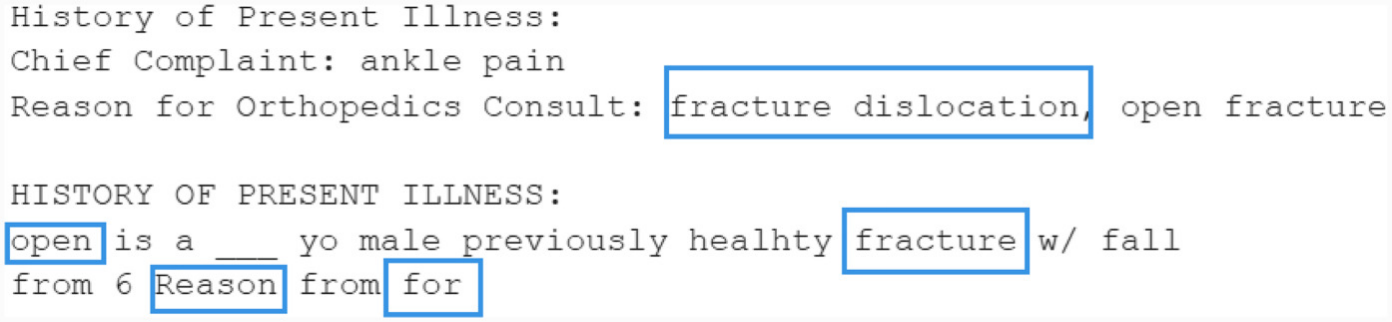
Example sentence generated by T5-base.

To further evaluate different PLMs in generating synthetic letters, we tested the T5 family models. The generated results for the same sentence are shown in Figure 12 and Supplementary Figures S16–S18.

T5-base performs the best among these tested models. However, the results are still not fully rational, as it generated “open is a ___ yo male.’. The other three models tend to use de-identification (DEID) tags to replace the masked words, as these tags are part of their corpora. Furthermore, the T5 family models may predict multiple words for each token, aligning with findings in Section 2.3.

All these four T5 family models perform worse than the encoder-only models. This is consistent with the findings from Micheletti et al. (78) that MLM models outperform CLM models in medical datasets.

### 4.3 Random masking: quantitative results

#### 4.3.1 Sentence-level quantitative results: encoder-only models

We first calculated representative quantitative metrics at the sentence level, matching the sample sentence used in Section 4.2. This approach allows for a better integration of quantitative and qualitative evaluations. Although SMOG is typically suited for medical datasets, it is less appropriate for sentence-level analysis, so the Flesch Reading Ease was used here. The results are presented in Table 1.

**Table 1:** Encoder-only models comparison at the sentence level (the “Baseline” without annotations was calculated by comparing the masked text to the original text).

Evaluation metric	Model evaluation			
	RoBERTa-base	medicalai/ClinicalBERT	Clinical-Longformer	Bio _ ClinicalBERT
ROUGE-1				
Generation performance	86.54	88.46	89.52	84.91
baseline	84.91	84.91	84.91	84.91
ROUGE-2				
Generation performance	74.51	78.43	79.61	73.08
baseline	73.08	73.08	73.08	73.08
ROUGE-L				
Generation performance	86.54	88.46	89.52	84.91
baseline	84.91	84.91	84.91	84.91
BERTScore F1				
Generation performance	0.81	0.83	0.84	0.85
baseline	0.79	0.65	0.79	0.65
METEOR				
Generation performance	0.87	0.88	0.90	0.86
baseline	0.85	0.85	0.85	0.85
Flesch Reading Ease				
Generation performance	10.24	18.70	9.22	16.67
baseline (original)	8.21	8.21	8.21	8.21
Baseline (mask)	16.67	16.67	16.67	16.67

Our objective is to generate letters that differ from the original while maintaining clinical semantics and structure. Thus, high ROUGE scores are not desired, as they indicate substantial word/string overlap. The BERTScore is particularly useful for assessing semantic similarity, while METEOR offers a comprehensive evaluation considering word forms and synonyms theoretically. Flesch Reading Ease, on the other hand, provides a direct measure of textual readability.

We observed that clinical-related encoder-only models generally outperform RoBERTa-base in qualitative evaluation (see Section 4.2). However, from the quantitative perspective, RoBERTa-base shows mediocre performance across most metrics except for the BERTScore. In contrast, Bio_ClinicalBERT, despite no word overlap in this sample sentence, achieves a reasonable clinical context and the highest BERTScore among the models. Both medicalai/Clinical BERT and Bio_ClinicalBERT excel in Flesch Reading Ease, likely because they tend to predict tokens with fewer syllables that preserve the original meaning.

Surprisingly, while METEOR is designed to closely reflect human evaluation, the BERTScore appears to be more consistent with our evaluation criteria. This trend was observed in other sample texts as well. *Synthetic texts with higher BERTScore and lower ROUGE scores are more aligned with our objectives*. It is likely because the BERTScore is calculated using word embeddings, which can capture deep semantic similarity more effectively. All evaluation results *meet or exceed the baseline, affirming the effectiveness of these four encoder-only models* in generating clinical letters.

#### 4.3.2 Sentence-level quantitative results: encoder–decoder models

The evaluations for the encoder–decoder models, as presented in Table 2, generally underperform on most metrics compared to encoder-only models, except for METEOR. Interestingly, while the Flesch Reading Ease scores suggest a minimal impact on readability, the BERTScores are at least 0.05 lower than the baseline, indicating major deviations from the original meaning. This is consistent with our qualitative observations that the outputs from encoder–decoder models are largely unintelligible.

**Table 2:** Encoder–decoder models comparison at the sentence level (the baseline without annotations was calculated by comparing the masked text to the original text).

Evaluation metric	Model evaluation			
	T5-base	Clinical-T5-base	Clinical-T5-scratch	Clinical-T5-Sci
ROUGE-1				
Generation performance	86.79	85.19	87.38	80.36
baseline	73.77	73.77	73.77	73.77
ROUGE-2				
Generation performance	75.00	71.70	75.25	69.09
baseline	63.33	63.33	63.33	63.33
ROUGE-L				
Generation performance	84.91	83.33	87.38	80.36
baseline	73.77	73.77	73.77	73.77
BERTScore F1				
Generation performance	0.44	0.40	0.45	0.40
baseline	0.50	0.50	0.50	0.50
METEOR				
Generation performance	0.85	0.83	0.83	0.82
baseline	0.85	0.85	0.85	0.85
Flesch Reading Ease				
Generation performance	8.21	8.21	19.71	8.21
baseline (original)	8.21	8.21	8.21	8.21
Baseline (mask)	8.21	8.21	8.21	8.21

Collectively, the quantitative and qualitative results demonstrate that ***encoder–decoder models are not well-suited for generating clinical letters***, as they fail to preserve the original narratives. These results also support the validity of using BERTScore as the primary evaluation metric, with other metrics serving as supplementary references. We also tested this on the entire dataset, which produced *consistent* results.

#### 4.3.3 Quantitative results on the full dataset: encoder-only models

Based on the findings above, we expect a higher BERTScore and a lower ROUGE score. We used the 0.4 masking ratio to illustrate the model comparison on the full dataset in Table 3. The other masking ratios show similar trends. Surprisingly, all encoder-only models this time showed comparable results, which contradicts our hypothesis that “Clinical-related” models would outperform base models. This suggests that *training on the clinical dataset has limited impact on the quality of synthetic letters*. This may be because most clinical-related tokens are preserved, with only the remaining tokens being eligible for masking. Consequently, the normal encoder-only models can effectively understand the context and predict appropriate words while preserving clinical information. This differs slightly from the sentence-level comparisons, likely because the evaluation of a single sentence cannot fully represent the overall results. Despite this, the BERTScore as a primary evaluation metric remains useful, as the correspondence between qualitative and quantitative evaluation is consistent, whether at the sentence or dataset level.

**Table 3:** Encoder-only models comparison on the full dataset with Masking Ratio 0.4 (the baseline was calculated by comparing the masked text to the original text).

Evaluation metric	Model evaluation			
	RoBERTa-base	medicalai/ClinicalBERT	Clinical-Longformer	Bio_ ClinicalBERT
ROUGE-1				
Generation performance	92.98	93.63	94.66	93.18
baseline	85.64	85.44	85.64	85.61
ROUGE-2				
Generation performance	86.10	87.42	89.50	86.50
baseline	74.96	74.64	74.96	74.92
ROUGE-L				
Generation performance	92.54	93.22	94.38	92.71
baseline	85.64	85.44	85.64	85.61
BERTScore F1				
Generation performance	0.91	0.90	0.92	0.90
baseline	0.82	0.63	0.82	0.63

We now explore how different *masking ratios* affect the quality of synthetic clinical letters. For each model, we generated data with masking ratios from 0.0 to 1.0, in increments of 0.1 (the masking ratios here refer only to the eligible tokens, as described in Section 3.4.3, and do not represent the actual overall masking ratio). Due to space limitations, we will present only the results for Bio_ClinicalBERT with a 0.2 increment here.

Table 4 presents that the higher masking ratio, the lower the similarity (metrics’ scores). As we expected, all evaluation values are higher than the baseline, but still below “1.” This means the model can understand the clinical context and generate understandable text. It is surprising that with a masking ratio of 1.0, the BERTScore increased from the baseline (0.29) to 0.63. Although this score is not very high, it still reflects that Bio_ClinicalBERT can generate clinical text effectively.

**Table 4:** Standard NLG metrics across different masking ratios using Bio_ClinicalBERT (the baseline was calculated by comparing the masked text to the original text).

Bio_ClinicalBERT	Masking ratio					
	1.0	0.8	0.6	0.4	0.2	0.0
ROUGE-1						
Generation performance	76.28	83.75	88.91	93.18	96.76	99.51
baseline	64.05	71.56	78.56	85.61	92.63	99.22
ROUGE-2						
Generation performance	62.60	70.77	78.81	86.50	93.42	99.02
baseline	51.72	57.88	65.38	74.92	86.27	98.61
ROUGE-L						
Generation performance	74.33	81.69	87.71	92.71	96.65	99.50
baseline	64.05	71.56	78.56	85.61	92.63	99.22
BERTScore						
Generation performance	0.63	0.75	0.83	0.90	0.95	0.99
baseline	0.29	0.39	0.50	0.63	0.79	0.98
METEOR						
Generation performance	0.70	0.80	0.87	0.93	0.97	1.00
baseline	0.66	0.72	0.78	0.85	0.92	0.99

In Supplementary Table S8, we calculated three readability metrics, which are mentioned in Section 3.5. None of these metrics showed significant differences from the original ones. However, it is strange that the SMOG and Flesh–Kincaid Grade are not always between the original baseline and masking baseline. When the masking ratio is high, the evaluation values even fall below both the masking and the original baseline. This may be because a *higher masking ratio leads to a lower valid prediction rate*. If the predicted words include many spaces or punctuation marks, the readability will decrease obviously.

In Supplementary Table S9, considering the perplexity, the masking baseline is very high, while the values for synthetic letters are close to the original ones. This indicates that the synthetic letters are useful for training clinical models. For information entropy, regardless of the masking ratio, it can *effectively preserve the amount of information*. As for subjectivity, since all the values are similar, we do not need to worry that the synthetic letters will be biased.

As shown in Table 5, inference time for the entire dataset consistently ranges between 3 and 4 h. However, it decreases with either very high or very low masking ratios. A mid-range masking ratio of approximately 0.6 results in longer inference times, likely because lower ratios reduce the number of masked tokens to process, while higher ratios provide less context, reducing the computational load. This lack of effective context also increases the invalid prediction rate. Conversely, with a masking ratio of “0,” even a small number of prediction errors can substantially affect the overall accuracy, as only a few tokens are masked.

**Table 5:** Inference time and invalid prediction rate across different masking ratios using Bio_ClinicalBERT.

Masking ratio	1.0	0.8	0.6	0.4	0.2	0.0
Inference time	3:12:05	3:28:56	3:33:26	3:25:16	3:13:26	3:01:11
Invalid prediction rate	0.72	0.47	0.34	0.28	0.25	0.37

### 4.4 Other masking strategies using Bio_ClinicalBERT

**Figure 13:**
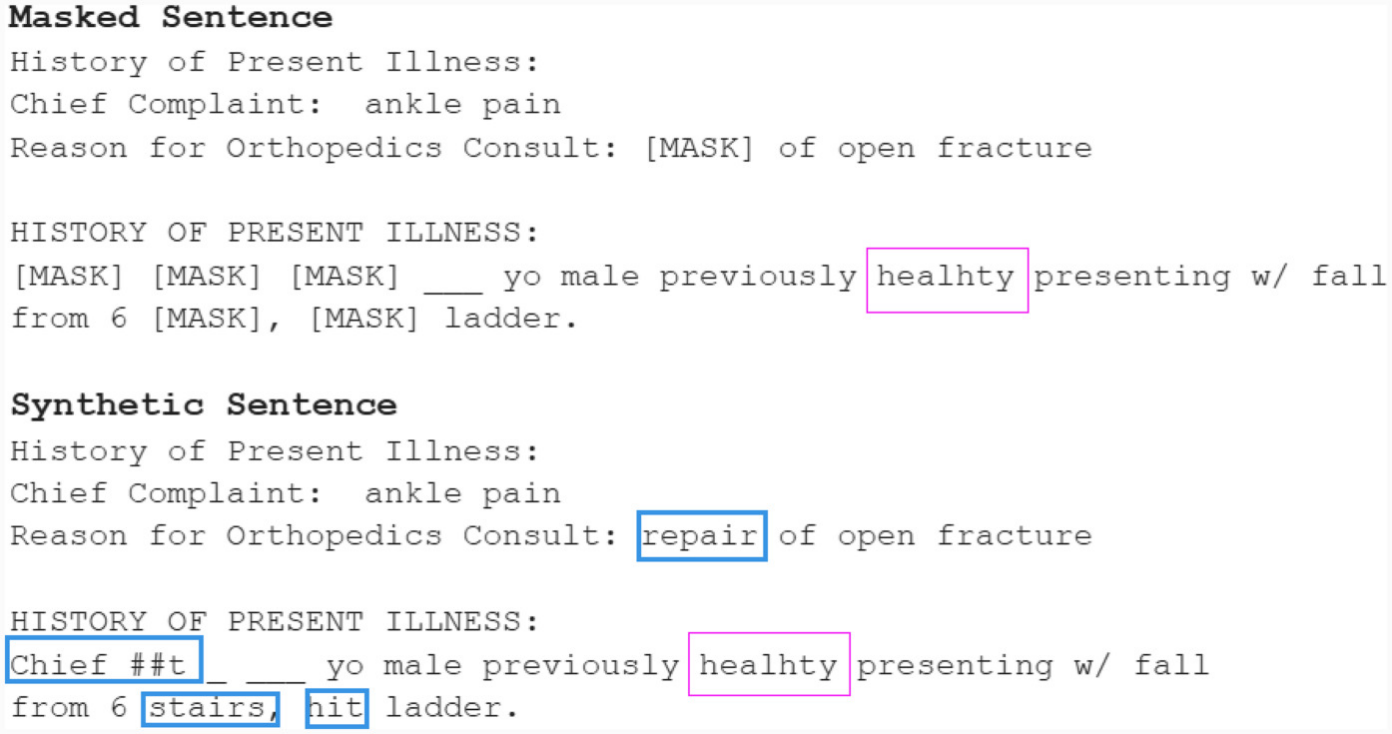
Example sentence 1 with different masked tokens.

There is a random selection when masking tokens at certain ratios. Masking different types of tokens will lead to different results, as shown in Figure 13 and Supplementary Figure S19. This variability is understandable since the encoder-only models use bidirectional attention, as mentioned in Section 2.3. *These models need to predict the masked tokens based on the context*. Therefore, it is necessary to experiment with different masking strategies based on the types of tokens. We used POS tagging and stopwords to observe how these strategies influence the quality of synthetic letters.

As discussed in Section 4.3, the BERTScore should be the primary evaluation metric for our objective. Additionally, the invalid prediction rate is useful for assessing the model’s ability to generate informative predictions, and ROUGE scores help evaluate literal diversity. Therefore, these quantitative metrics, calculated using different masking strategies, are shown in this section. Similar to Section 4.3, we experimented with different masking ratios calculated from the eligible tokens (masked tokens divided by eligible tokens). The ratios are increased in increments of 0.1, ranging from 0.0 to 1.0. Due to space constraints, only metrics with increments of 0.2 are shown here. A comparison with the same actual masking ratio (masked tokens divided by total tokens in the text) are also presented in this subsection.

#### 4.4.1 Masking only nouns

Nouns often correspond to personally identifiable information (PII), so masking nouns can serve as a verification step for de-identification.

As shown in Supplementary Table S10, the fewer nouns we mask, the better all these metrics perform. This trend is consistent with random masking. When the noun masking ratio is 1.0, meaning that all nouns are masked, the BERTScore increases from a baseline of 0.70 to 0.89. This means that the *model predicted meaningful nouns*. A similar trend is observed for the ROUGE scores. All evaluations are higher than the baseline but lower than “1.” However, ROUGE scores show a smaller improvement than BERTScore. This may be because the model generates synonyms or paraphrases that retain the original meaning. As the noun masking ratio increases from 0.0 to 1.0, the BERTScore decrease from 0.99 to 0.89, indicating a significant decrease.

Therefore, to generate synthetic clinical letters that are distinguishable but still retain the original clinical information, we can only partially mask nouns (around 0.8 masking ratio). It helps maintain balanced evaluation scores. When all nouns are masked, the quality of synthetic letters deteriorates, with the BERTScore falling below 0.9 and the invalid prediction rate increasing to 0.37.

#### 4.4.2 Masking only verbs

Masking only verbs also helps identify which token types are appropriate for masking to achieve our objective. While verbs are essential to describing clinical events, some can still be inferred from context. Therefore, *masking verbs may have a slight effect on the quality of synthetic clinical letters, but it can also introduce some variation*.

Supplementary Table S11 shows a similar trend for masking verbs as observed with other masking strategies in standard NLG metrics. However, it is surprising that as the masking ratio increases, both the invalid prediction rate and NLG metrics decrease. This phenomenon can be attributed to two main reasons. First, the model seems to prioritize predicting meaningful tokens (rather than punctuation, spaces, etc.) to generate coherent sentences. Contextual relevance is only considered after the sentence structure is complete. This may be due to the important role of verbs in sentences. Second, the original raw data may contain fewer verbs than nouns. Therefore, the number of actual masked tokens changes slightly when verbs are masked, making the model less sensitive to them. This is also reflected in BERTScore. If all verbs are masked, the BERTScore remains high at 0.95, whereas if all nouns are masked, the BERTScore drops to 0.89.

#### 4.4.3 Masking only stopwords

As mentioned in Section 3.4.3, masking stopwords aims to reduce noise for model understanding while introducing variation in synthetic clinical letters. Supplementary Table S12 shows that *masking only stopwords follows a similar trend to random masking*, where a higher masking ratio leads to lower ROUGE Score and BERTScore. Additionally, the invalid prediction rate is at its lowest with a medium masking ratio. This is because higher masking ratios always result in more information loss. On the other hand, lower masking ratios lead to fewer tokens being masked, which makes small prediction errors more influential. The results show an overall low Invalid Prediction Rate and high BERTScore, indicating that *stopwords have only a limited influence on the model’s understanding of context*. This is not because the original raw letters contain very few stopwords. In fact, there are even more stopwords than nouns and verbs, as seen in sample texts.

#### 4.4.4 Comparison of identical actual masking ratios

To further observe how different masking strategies influence the generation of clinical letters, we compared the results using the same actual masking ratios but with different strategies. In other words, the number of masked tokens is fixed, so the only variable is *the type of tokens being masked*. Supplementary Table S13 shows the results with a 0.04 actual masking ratio, and Table 6 shows the results with a 0.1 actual masking ratio.

**Table 6:** Quantitative comparisons of 0.1 actual masking ratio (the baseline was calculated by comparing the masked text to the original text).

Bio_ClinicalBERT	Nouns masking (1.0)	Stopwords masking (0.6)	Random masking (0.3)
ROUGE-1			
Generation performance	93.29	96.56	95.10
baseline	88.13	89.04	89.16
ROUGE-2			
Generation performance	86.71	92.53	90.17
baseline	78.32	79.99	80.44
ROUGE-L			
Generation performance	93.00	96.23	94.86
baseline	88.13	89.04	89.16
BERTScore			
Generation performance	0.89	0.95	0.93
baseline	0.70	0.71	0.71
Invalid prediction rate			
Generation performance	0.37	0.20	0.26

As we can see, masking only stopwords achieved the highest BERTScore and lowest invalid prediction rate. Therefore, stopwords have little influence on the overall meaning of the text, which is consistent with our earlier findings. Additionally, masking nouns and verbs performed worse than random masking. Therefore, if we want to preserve the original meaning, we cannot mask too many nouns and verbs.

#### 4.4.5 Hybrid masking

After comparing different strategies with the same actual masking ratio, we explored hybrid masking strategies and compared them with other strategies at the same actual ratio. The results are presented in Supplementary Table S14. The first three columns have the same actual masking ratio. Masking only stopwords achieved the strongest performance among these strategies. However, when nouns were also masked along with stopwords, the performance decreased, as masking nouns negatively affect the results. Despite this, it still performed better than random masking, indicating that stopwords have a greater influence than nouns. Next, we compared the last two columns. If 0.5 of nouns and 0.5 of stopwords were masked, adding an additional 0.5 of masked verbs led to worse performance, showing that verbs also negatively influence the model’s performance.

#### 4.4.6 Comparison with and without (w/o) entity preservation

To further explore whether keeping entities is useful for our task, we compared our results with a baseline that does not retain any entities. The baseline was trained with four epochs of fine-tuning on our dataset. Specifically, 0.4 of nouns from all tokens were randomly masked during baseline training. In contrast, in our experiments, only eligible tokens—excluding clinical information—were selected for masking. The comparisons are shown in Table 7.

**Table 7:** Comparison with and without entity preservation using Bio_ClinicalBERT.

Bio_ClinicalBERT	With entity preservation (0.4 nouns masking)	With entity preservation (0.3 random masking)	Without entity preservation (0.4 nouns masking)
ROUGE-1	97.62	95.10	97.31
ROUGE-2	95.12	90.17	94.46
ROUGE-L	97.56	94.86	93.71
BERTScore	0.96	0.93	0.91

As we can see, when 0.4 nouns were masked while preserving entities, the models performed much better than those without any entity preservation. Interestingly, when we randomly masked 0.3 while preserving entities, the model achieved lower ROUGE-1 and ROUGE-2 scores but higher ROUGE-L and BERTScores compared to models without entity preservation. This trend is consistent across different settings. This suggests that models preserving entities show less overlap with the original text, while they can retain the original narrative better. Additionally, the higher ROUGE-L score suggests that the step of preserving document structure is indeed effective.

These results also confirm our initial hypothesis that, for our objective—generating clinical letters that can keep the original meaning while adding some variety—retaining entities is much more effective than just fine-tuning the model. Moreover, this approach can effectively preserve useful information while avoiding overfitting.

### 4.5 Downstream NER task

To further evaluate whether synthetic letters have the potential to replace the original raw letters, particularly in the domains of clinical research and model training, a downstream NER task was implemented. Two spaCy NER models were trained separately on original raw letters and synthetic letters. Specifically, the synthetic letters were generated with 0.3 random masking while preserving entities.

As shown in Table 8, spaCy models trained on original and synthetic letters showed similar evaluation scores. They even achieved F1 scores comparable to ScispaCy’s score of 0.843. Therefore, the unmasked context appears to have minimal influence on model understanding. Consequently, *our synthetic letters can be used in NER tasks to replace real-world clinical letters, thereby further protecting sensitive information*.

**Table 8:** Comparisons on downstream NER task.

Metric	spaCy trained on original letters	spaCy trained on synthetic letters	Performance Delta (Δ)
F1 Score	0.855	0.853	−0.002
Precision	0.865	0.863	−0.002
Recall	0.846	0.843	−0.003

### 4.6 Clinical evaluation

#### 4.6.1 Clinical semantic preservation

As mentioned in Section 3.5.4, we used BioGPT with a random masking ratio of 0.3 to evaluate the integrity of clinical narrative preservation. As shown in Table 9, the mean similarity score reaches 0.98, which is slightly higher than the score obtained using the BERTScore metric. This may be because BioGPT evaluates semantic similarity from a clinical perspective. Additionally, such a high score suggests that the synthetic clinical letters can potentially serve as replacements for the original ones.

**Table 9:** Results of clinical semantic preservation evaluation using BioGPT.

Metric	Max score	Min score	Mean score	Std deviation	Evaluation set size
Value	0.9996	0.9037	0.9896	0.0147	204

#### 4.6.2 Expert-simulated evaluation of clinical quality

As mentioned in Section 3.5.4, we prompted GPT-3.5-Turbo to simulate a clinical expert and evaluate clinical soundness and narrative coherence. The masked letters (with text replaced by “<mask>”) continued to serve as a baseline. The results are shown in Table 10

**Table 10:** Expert-simulated evaluation results.

Metric	Avg.	Max.	Min.	Std.
Clinical soundness				
Baseline (original)	0.766	1.0	0.5	0.11
Baseline (masked)	0.611	1.0	0.5	0.147
Generation performance	0.604	1.0	0.5	0.145
Narrative coherence				
Baseline (original)	0.664	0.8	0.3	0.082
Baseline (masked)	0.418	0.7	0.2	0.168
Generation performance	0.460	0.7	0.2	0.177

**Clinical soundness:** The average clinical soundness score of the generated letters (0.604) is slightly lower than that of the original letters (0.766). Surprisingly, it is even lower than the score of the masked letters (0.611). We further identified all cases where the generated letters scored lower than the masked ones in clinical soundness. These cases account for 14% (29 out of 204) of the processed letters. One possible explanation is that Bio_ClinicalBERT occasionally produces hallucinatory content, which may obscure or distort the original clinical semantics. However, in the majority of cases, the generated letters achieve clinical soundness scores comparable to the masked letters and close to the original ones—demonstrating the overall potential of our synthetic letters to replace real ones.

**Narrative coherence:** As expected, the narrative coherence score of the generated letters (0.460) is slightly lower than that of the original ones (0.664), but higher than that of the masked letters (0.418). These results further support the feasibility of using synthetic letters as substitutes for real clinical letters.

### 4.7 Post-processing results

#### 4.7.1 Filling in the blanks

**Figure 14:**
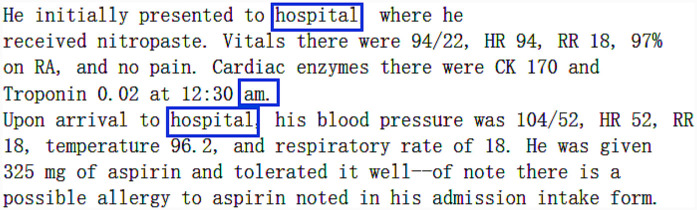
Post-processing results with BERT-Base.

One example text without post-processing is shown in Supplementary Figure S20. After filling in the blanks, the results with BERT-base and Bio_ClinicalBERT are shown in Figure 14 and Supplementary Figure S21, respectively. We can see that both models can partially achieve the goal of making the text more complete. However, neither of them created a coherent story to fill in these blanks. They just used general terms like “hospital” and “clinic.” Perhaps other decoder-only models, more suitable for generating stories like GPT, could perform better and should be explored in the future.

#### 4.7.2 Spelling correction

Supplementary Figure S22 shows that if the incorrect words are masked, the models may be able to correct the misspelled tokens by predicting them. However, the masking process is random. Additionally, sometimes the predicted words will be incorrect because some models tokenise the sentence into word-pieces. Therefore, a post-processing step is necessary for correcting spelling.

As shown in Supplementary Figure S23, tooltik “TextBlob” (93) can successfully correct misspelled words (“healhty”) in our sample text. However, if clinical entities are not preserved during the pre-processing step, “TextBlob” (93) may misidentify some clinical terms as spelling errors. This may be because “TextBlob” (93) was developed on the general corpus rather than a clinical one. Additionally, its corrections are limited to the word level and do not consider any context. Therefore, if words are misspelled deliberately, they could be processed incorrectly. Thus, *developing a clinical misspelling correction toolkit is a promising* research direction in the future.

### 4.8 Discussion

We found that different masking strategies result in notable differences in model performance. To enhance the practical applicability of our research, we provide a guideline for selecting appropriate masking strategies for different scenarios, as presented in Table 11.

**Table 11:** Priority-based masking guidelines.

Priority	Note	Suggested masking strategy	Application scenarios
Diversity first	To improve the model’s generalisation	Random masking (primarily), clinical terms masking (limited)	Basic clinical model pre-training; data augmentation
Clinical soundness first	The synthetic letters should satisfy clinical factuality	Keep clinical terms (complete); stopwords masking (extensive); verbs/nouns masking	Clinical education; clinical QA model training; clinical model fine-tuning
Privacy first	To prevent PHI disclosure and mitigate privacy reconstruction through adversarial attacks	Private tokens masking (complete); nouns masking (extensive); verbs/stopwords masking (medium)	Building open-source datasets; commercial deployment

As mentioned earlier, we observe that when most clinical terms are preserved, fine-tuning the model may not be necessary. In terms of clinical evaluation, hallucinated content was found to negatively affect clinical soundness, suggesting that retrieval-augmented generation (RAG) or integration with a clinical knowledge graph may be beneficial for future improvements. Further exploration is also needed—such as dynamic vocabulary construction—to better handle clinical abbreviations and novel terms. Our synthetic framework for clinical letters did not show any notable negative effects on narrative coherence or semantic preservation, and the high performance in downstream NER tasks further supports the feasibility of using synthetic letters as substitutes for original ones. Although filling in blanks and correcting spelling errors are essential for improving text quality, mitigating errors in processing rare clinical terms remains a major challenge, as previously discussed.

## 5 Conclusions and future work

### 5.1 Key findings

These results provided some useful findings in generating clinical letters, including

• **Encoder-only models generally perform much better** in clinical-letter masking and generation tasks, which is consistent with a very recent study by Micheletti et al. (78). When clinical information is preserved, **base encoder-only models perform comparably to clinical-related models**.
• To generate clinical letters that preserve clinical narrative while adding variety, **BERTScore should be the primary evaluation metric**, with other metrics serving as supporting references. This is because BERTScore focuses more on semantic rather than literal similarity, and it is consistent with qualitative assessment results.
• **Different types of masked tokens influence** the quality of synthetic clinical letters. Stopwords exert a positive impact, while nouns and verbs exert negative impacts.
• For our objective, preserving useful tokens is more effective than just fine-tuning the model without preserving any entities.
• The unmasked context has minimal influence on the models’ understanding. As a result, the synthetic letters can be effectively used in the downstream NER task to replace original real-world letters.
• The synthetic letters largely preserve the consistency and coherence of clinical narratives from the original letters. However, Bio_ClinicalBERT occasionally generates hallucinated content, which may negatively impact clinical soundness and factuality.

### 5.2 Limitations

Although the strategies mentioned above help generate diverse, de-identified synthetic clinical letters, there are still some limitations in applying this method generally.

• **Challenges in the dataset:** Since these clinical letters are derived from the real world, certain issues are inevitable. For example, there may be spelling errors in the dataset. In note_id “10807423-DS-19,” the word “healthy” is misspelled as “healhty.” Such errors can negatively impact the usability of the synthetic text. Additionally, some polysemous words may cause contextual ambiguity. For instance, the word “back” can refer to an anatomical entity (e.g., the back of the body), or be used as an adverb.
• **Data volume:** Due to the difficulty in collecting annotated data, only 204 clinical letters were included in our research. This limited sample size may not be sufficiently representative, which could restrict the generalizability of our findings to a broader scenario. Moreover, the data we used were already de-identified. Although we considered de-identification and took steps to mask all private information, the effectiveness of these approaches cannot be thoroughly evaluated, as we do not have access to sensitive datasets.
• **Evaluation metrics:** In this paper, we primarily used BERTScore as our main evaluation metric, while also incorporating other metrics such as ROUGE and readability metrics. However, there is currently no comprehensive evaluation framework that can assess all aspects simultaneously, including maintaining the original meaning, diversity, readability, clinical soundness, and even privacy protection effectiveness.
• **Clinical knowledge understanding:** While the model can often preserve clinical entities and generate contextually reasonable tokens, it sometimes makes comprehension errors. For example, in a context where “LLE” (“left lower extremity”) is used, Bio_ClinicalBERT incorrectly predicts the nearby masked token as “R ankle” (“right ankle”). In this case, the model fails to accurately capture the side clinical knowledge. Other challenges lie in handling long-tail phenomena and understanding abbreviated expressions, which are common in clinical language. Although spell correction techniques are explored in our project, distinguishing between a genuinely novel term and a simple misspelling remains difficult.
• **Computing resources:** Due to resource limitations, we explored a limited range of language generation models. Alternative architectures—such as enhanced decoder-only models—may be more suitable for our task.

### 5.3 Future work

Based on the limitations mentioned above, we outlined some potential directions to further explore:

• **Test on more clinical datasets:** To further evaluate the effectiveness of these masking strategies, more annotated clinical letters should be tested to assess system generalization.
• **Assess de-identification performance:** A quantitative metric for de-identification evaluation should be included in the future. Non-anonymous synthetic datasets can be used to evaluate the de-identification process, so that this system can be applied directly to real-world clinical letters in the future.
• **GRPO-based reinforcement learning:** The group relative policy optimization (GRPO) algorithm, as proposed in DeepSeek (94), has the potential to effectively balance multiple objectives, including clinical soundness, semantic integrity, textual diversity, and de-identification quality.
• **Evaluation benchmark:** A new metric suitable for our task should be developed. Specifically, this metric should consider both similarity and diversity. Weighting parameters for each dimension could be useful and can be obtained through neural networks. For evaluating clinical soundness, it is necessary to invite more clinicians to assess the synthetic letters based on multiple dimensions (77). Furthermore, mapping from clinical letters to their quality scores can be learned using deep learning.
• **Balancing knowledge from both clinical and general domains:** Although there are numerous clinical-related encoder-only models, only a few can effectively integrate clinical and general knowledge. Xie et al. (95) demonstrated that mixing the clinical dataset with the general dataset in a certain proportion can help the model better understand clinical knowledge. Therefore, a new BERT-based model should be trained from scratch using both clinical and general domain datasets.
• **Synonymous substitution:** We focused on exploring the range of eligible tokens for masking. Additionally, a masking strategy similar to BERT’s can be integrated with our results (58). Specifically, we can select certain tokens to mask, some to retain, and replace others with synonyms. This approach can further enhance the variety of synthetic clinical letters. Moreover, the retained clinical entities can also be substituted using entity linking to SNOMED CT.
• **Spelling correction:** As mentioned in Section 4.7, very few toolkits are available for spelling correction in the clinical domain. Standard spelling correction tools may misidentify clinical terms as misspelled words. Therefore, it is necessary to develop a specialized spell-checking tool adapted to the clinical domain.

## Data Availability

The data used in this study are from the publicly available MIMIC database (Medical Information Mart for Intensive Care), accessible to qualified researchers who complete the PhysioNet credentialing process and agree to the Data Use Agreement (https://physionet.org/content/mimiciv/2.2/).

## Footnotes

^1^We report our extended solid investigation and outcomes based on our preliminary workshop paper findings (96).

^2^ https://spacy.io/

^3^ https://allenai.github.io/scispacy/

^4^LT3 achieved significant improvements over the best-performing T5 model (T5 base) in label-to-text generation, achieving improvements of up to 6.5 BLEU points and 0.02 in the BERTScore. Unfortunately, when we tried applying B2SD to generate clinical letters, the results were somehow disappointing. This may be due to the length of clinical letters. B2SD consumes a lot of time on long text generation. Despite this, it still shows great potential in generating clinical data.

^5^ https://spacy.io/

^6^We define a significant difference as a change of 1 SMOG grade, 1 Flesch–Kincaid Grade Level, or 10 points in Flesch Reading Ease, as these thresholds approximately correspond to a shift of one grade level or readability tier.

^7^ https://allenai.github.io/scispacy/

^8^ https://spacy.io/

## Supplementary material

**Supplementary Figure S1**

An example of LT3 (76).

**Supplementary Figure S2**

An input example of conditional text generation (75).

**Supplementary Figure S3**

Workflow of discharge summary generation using clinical guidelines.

**Supplementary Figure S4**

Workflow of MLM and CLM comparison in text generation.

**Supplementary Figure S5**

Text excerpt from the original letter (6–8) (“note_id”: “17656866-DS-6”).

**Supplementary Figure S6**

Sample text from original letters (6–8) (“note_id”: “10807423-DS-19”).

**Supplementary Figure S7**

Sentence fragment exceeding token limit (6–8) (“note_id”: “10807423-DS-19”).

**Supplementary Figure S8**

Example sentence for feature extraction (see Supplementary Table S5 for Extracted Features).

**Supplementary Figure S9**

Training process for spaCy NER model.^9^

**Supplementary Figure S10**

An example of masking and generating.

**Supplementary Figure S11**

Extracted structure from the example sentence.

**Supplementary Figure S12**

Example sentence generated by medicalai/ClinicalBERT.

**Supplementary Figure S13**

Example sentence generated by Clinical-Longformer.

**Supplementary Figure S14**

Example sentence generated by RoBERTa-base.

**Supplementary Figure S15**

Example sentence generated by GPT-4o.

**Supplementary Figure S16**

Example sentence generated by Clinical-T5-Base.

**Supplementary Figure S17**

Example sentence generated by Clinical-T5-Scratch.

**Supplementary Figure S18**

Example sentence generated by Clinical-T5-Sci.

**Supplementary Figure S19**

Example sentence 2 with different masked tokens.

**Supplementary Figure S20**

Example sentence before post-processing (6–8) (“note_id”: “16441224-DS-19”).

**Supplementary Figure S21**

Post-processing results with Bio_ClinicalBERT.

**Supplementary Figure S22**

Spelling correction by masking and generating (6–8) (“note_id”: “10807423-DS-19”).

**Supplementary Figure S23**

Spelling correction (6–8) (“note_id”: “10807423-DS-19”).

**Supplementary Table S1**

Encoder-only models and their fine-tuned datasets.

**Supplementary Table S2**

The T5 family models used in our work.

**Supplementary Table S3**

Extracted entities and their details.

**Supplementary Table S4**

Comparison of tokenization methods for different LMs on sentence “Patient is a ___ yo male previously healthy presenting w/fall from 6 feet, from ladder.”

**Supplementary Table S5**

Example: summary of feature extraction operations and extracted features.

**Supplementary Table S6**

Relation between chunk sizes and model inference time, average token number.

**Supplementary Table S7**

Annotated entities extracted from the example sentence (they should be preserved from masking).

**Supplementary Table S8**

Readability metrics across different masking ratios using Bio_ClinicalBERT (the Baseline without annotations was calculated by comparing masked text to the original text).

**Supplementary Table S9**

Advanced text quality metrics across different masking ratios using Bio_ClinicalBERT (the baseline without annotations was calculated by comparing masked text to the original text).

**Supplementary Table S10**

Quantitative comparisons of noun masking ratio (the “Baseline” was calculated by comparing masked text to the original text).

**Supplementary Table S11**

Quantitative comparisons of verb masking ratios (the “Baseline” was calculated by comparing masked text to the original text).

**Supplementary Table S12**

Quantitative comparisons of stopword masking ratios (the “Baseline” was calculated by comparing masked text to the original text).

**Supplementary Table S13**

Quantitative comparison of different masking strategies at a 0.04 actual masking ratio (the “Baseline” was calculated by comparing masked text to the original text).

**Supplementary Table S14**

Quantitative comparisons for hybrid masking (the baseline was calculated by comparing masked text to the original text).
