# Supplementary material for: Synthetic4Health: generating annotated synthetic clinical letters
Source: Front Digit Health. 2025 May 30;7:1497130. doi: 10.3389/fdgth.2025.1497130 (PMC12163008; doi:10.3389/fdgth.2025.1497130)
Supplement: Supplementary file 1 [file Datasheet1.pdf]

Synthetic4Health: Generating Annotated Synthetic Clinical Letters

Libo Ren [1], Samuel Belkadi [2], Lifeng Han [1,3*], Warren Del-Pinto [1], and Goran Nenadic [1]

[1] *University of Manchester, Oxford Rd, Greater Manchester, UK*
[2] *Department of Engineering, University of Cambridge, UK*
[3] *LIACS & LUMC, Leiden University, NL*

Correspondence*:
Corresponding Author
l.han@lumc.nl — lifeng.han@manchester.ac.uk

1 SUPPLEMENTARY MATERIAL

Model	Fine-tuned Dataset
Bio_Clinical BERT (Alsentzer et al., 2019)	MIMIC-III
medicalai/ClinicalBERT (Wang et al., 2023)	A large corpus of 1.2B words of diverse diseases
RoBERTa-base (Zhuang et al., 2021)	General Dataset (including BookCorpus, English Wikipedia, CC-News, OpenWebText, and Stories)
Clinical-Longformer (Li et al., 2023)	MIMIC-III

Supplementary Table 1. Encoder-Only Models and Their Fine-tuned Datasets

Model	Pre-trained Dataset	Weight Initialisation Method
T5-base (Raffel et al., 2020)	Colossal Clean Crawled Corpus (C4)	Randomly Initialised
Clinical-T5-Base (Eric and Johnson, 2023; Goldberger et al., 2000)	MIMIC-III, MIMIC-IV	Initialised from T5-Base
Clinical-T5-Sci (Eric and Johnson, 2023; Goldberger et al., 2000)	PubMed Abstracts, PubMed Central	Initialised from SciFive
Clinical-T5-Scratch (Eric and Johnson, 2023; Goldberger et al., 2000)	MIMIC-III, MIMIC-IV	Randomly Initialised

Supplementary Table 2. The T5 Family Models used in our work

REFERENCES

Alsentzer, E., Murphy, J., Boag, W., Weng, W.-H., Jindi, D., Naumann, T., et al. (2019). Publicly available clinical bert embeddings. In *Proceedings of the 2nd Clinical Natural Language Processing Workshop*. 72–78

[Dataset] Eric, L. and Johnson, A. (2023). Clinical-T5: Large Language Models Built Using MIMIC Clinical Text. PhysioNet. doi:10.13026/rj8x-v335

Entity	Start	End	Concept ID
fall	571	575	161898004
LLE	621	624	32153003
eversion	636	644	4196002
open fracture	660	673	397181002
dislocation	674	685	87642003
head strike	694	706	82271004
LOC	710	713	419045004
neck pain	722	731	81680005
back pain	733	742	161891005
chest pain	744	754	29857009
abd pain	756	765	21522001
pelvic	774	780	30473006
thigh pain	784	794	78514002
conscious sedation	833	851	314271007
vitals	874	880	118227000
neurovascular symptoms	963	986	308921004

Supplementary Table 3. Extracted Entities and Their Details

Operation / Model	Tokenization Method	Tokenized Output
Feature Extraction	Word Tokenization	[‘Patient’, ‘is’, ‘a’, ‘___’, ‘yo’, ‘male’, ‘previously’, ‘healthy’, ‘presenting’, ‘w’, ‘fall’, ‘from’, ‘6’, ‘feet’, ‘,’, ‘from’, ‘ladder’, ‘.]
medicalai / ClinicalBERT	Subword-Enhanced Word-Piece	[‘patient’, ‘is’, ‘a’, ‘_’, ‘_’, ‘_’, ‘yo’, ‘male’, ‘previously’, ‘healthy’, ‘presenting’, ‘w’, ‘/’, ‘fall’, ‘from’, ‘6’, ‘feet’, ‘,’, ‘from’, ‘la’, ‘##dder’, ‘.]
BERT-base, Bio_ClinicalBERT	Standard Word-Piece	['patient', 'is', 'a', '_', '_', '_', 'yo', 'male', 'previously', 'healthy', 'presenting', 'w', '/', 'fall', 'from', '6', 'feet', ',', 'from', 'ladder', '.']
Clinical-Longformer, RoBERTa	Detailed Word-Piece	['Pat', 'ient', 'Ġis', 'Ġa', 'Ġ___', 'Ġyo', 'Ġmale', 'Ġpreviously', 'Ġhealthy', 'Ġpresenting', 'Ġw', '/', 'Ġfall', 'Ġfrom', 'Ġ6', 'Ġfeet', ',', 'Ġfrom', 'Ġladder', '.']
T5 Family (T5 base, SCI T5, Clinical T5)	Sentence-Piece	[‘__Patient’, ‘__is’, ‘__’, ‘a’, ‘__’, ‘_’, ‘_’, ‘_’, ‘__’, ‘y’, ‘o’, ‘__male’, ‘__previously’, ‘__healthy’, ‘__’, ‘presenting’, ‘__’, ‘w’, ‘/’, ‘__fall’, ‘__from’, ‘__6’, ‘__feet’, ‘,’, ‘__from’, ‘__ladder’, ‘.’]

Supplementary Table 4. Comparison of Tokenization Methods for Different LMs
on Sentence ___
”Patient is a yo male previously healthy presenting w/ fall from 6 feet, from
ladder.”

Goldberger, A. L., Amaral, L. A. N., Glass, L., Hausdorff, J. M., Ivanov, P. C., Mark, R. G., et al. (2000). Physiobank, physiotoolkit, and physionet. *Circulation* 101, e215–e220. doi:10.1161/01.CIR.101.23.e215

Li, Y., Wehbe, R. M., Ahmad, F. S., Wang, H., and Luo, Y. (2023). A comparative study of pretrained language models for long clinical text. *Journal of the American Medical Informatics Association* 30, 340–347

Raffel, C., Shazeer, N., Roberts, A., Lee, K., Narang, S., Matena, M., et al. (2020). Exploring the limits of transfer learning with a unified text-to-text transformer. *Journal of Machine Learning Research* 21, 1–67

**Feature Extraction Operation**	**Extracted Features**
Structure Extraction	Discharge Medication:
Privacy Information Identification	Jone (PERSON)<br>06/03/2010 (DATE)<br>Postal Code: M16 3JE
Medical Terminology Recognition	Deep Vein Thrombosis (PROBLEM)<br>DVT (PROBLEM)<br>enoxaparin (TREATMENT)<br>the syringe (TREATMENT)<br>RX enoxaparin (TREATMENT)
Special Patterns Observed in Sample Text	'40 mg/0.4 mL' (122, 134)<br>'40 mg/0.4 mL ' (284, 297)<br>'#14' (323, 327)<br>'0' (344, 346)
POS Tagging	'Jone', 'PROPN'<br>'is', 'AUX'<br>'living', 'VERB'<br>'in', 'ADP'<br>*(Partial List)*

Supplementary Table 5. Example: Summary of Feature Extraction Operations and Extracted Features

**max_lines**	**10**	**20**	**30**	**35**	**40**	**41**	**42**	**45**	**50**
**Inference Time (min)**	13:47	8:10	6:44	5:24	5:10	5:01	5:12	5:54	6:05
**Average Tokens Per Chunk**	51.59	90.23	131.26	136.55	144.34	146.43	146.43	146.43	146.43

Supplementary Table 6. Relation between Chunk Sizes and (Model Inference Time, Average Token Number)

**Entity**	**Start**	**End**	**Concept ID**
ankle pain	411	421	247373008
open fracture	468	481	397181002
fall	571	575	161898004

Supplementary Table 7. Annotated Entities Extracted from the Example Sentence (They should be preserved from masking)

Wang, G., Liu, X., Ying, Z., Yang, G., Chen, Z., Liu, Z., et al. (2023). Optimized glycemic control of type 2 diabetes with reinforcement learning: a proof-of-concept trial. *Nature Medicine* 29, 2633–2642

Zhuang, L., Wayne, L., Ya, S., and Jun, Z. (2021). A robustly optimized BERT pre-training approach with post-training. In *Proceedings of the 20th Chinese National Conference on Computational Linguistics*, eds. S. Li, M. Sun, Y. Liu, H. Wu, K. Liu, W. Che, S. He, and G. Rao (Huhhot, China: Chinese Information Processing Society of China), 1218–1227

Bio_ClinicalBERT	Masking Ratio					
	1.0	0.8	0.6	0.4	0.2	0.0
**SMOG**						
Generation Performance	8.91	9.18	9.50	9.79	10.00	10.13
Baseline (Original)	10.16	10.15	10.15	10.15	10.15	10.15
Baseline (Mask)	9.04	9.29	9.52	9.74	9.95	10.13
**Flesch Reading Ease**						
Generation Performance	63.77	63.44	61.41	59.54	58.06	57.02
Baseline (Original)	56.85	56.87	56.87	56.87	56.87	56.87
Baseline (Mask)	70.11	67.39	64.75	62.15	59.62	57.13
**Flesch-Kincaid Grade**						
Generation Performance	7.32	7.70	8.24	8.66	9.01	9.22
Baseline (Original)	9.26	9.26	9.26	9.26	9.26	9.26
Baseline (Mask)	7.41	7.79	8.16	8.52	8.87	9.22

Supplementary Table 8. Readability Metrics Across Different Masking_Ratios Using Bio ClinicalBERT (The Baseline without annotations was calculated by comparing masked text to the original text)

Bio_ClinicalBERT	Masking Ratio					
	1.0	0.8	0.6	0.4	0.2	0.0
**Perplexity**						
Generation Performance	2.24	2.32	2.31	2.30	2.29	2.29
Baseline (Original)	2.22	2.28	2.28	2.28	2.28	2.28
Baseline (Mask)	250.37	65.42	24.29	8.95	4.03	2.39
**Information Entropy**						
Generation Performance	5.46	5.80	5.92	5.96	5.98	5.98
Baseline (Original)	5.98	5.98	5.98	5.98	5.98	5.98
Baseline (Mask)	4.51	4.93	5.29	5.60	5.85	5.97
**Subjectivity**						
Generation Performance	0.32	0.32	0.32	0.32	0.33	0.33
Baseline (Original)	0.33	0.33	0.33	0.33	0.33	0.33
Baseline (Mask)	0.41	0.39	0.38	0.37	0.35	0.33

Supplementary Table 9. Advanced Text Quality Metrics Across Different Masking_Ratios Using Bio ClinicalBERT (The Baseline without annotations was calculated by comparing masked text to the original text)

Bio_ClinicalBERT	Nouns Masking Ratio					
	1.0	0.8	0.6	0.4	0.2	0.0
**ROUGE-1**						
Generation Performance	93.29	95.16	96.48	97.62	98.66	99.51
Baseline	88.13	90.79	92.98	95.19	97.39	99.22
**ROUGE-2**						
Generation Performance	86.71	90.29	92.84	95.12	97.28	99.02
Baseline	78.32	82.92	86.79	90.82	95.01	98.61
**ROUGE-L**						
Generation Performance	93.00	94.96	96.35	97.56	98.64	99.50
Baseline	88.13	90.79	92.98	95.19	97.39	99.22
**BERTScore**						
Generation Performance	0.89	0.92	0.94	0.96	0.98	0.99
Baseline	0.70	0.76	0.81	0.86	0.92	0.98
**Invalid Prediction Rate**						
Generation Performance	0.37	0.34	0.33	0.32	0.32	0.37

Supplementary Table 10. Quantitative Comparisons of Nouns Masking Ratios (The 'Baseline' was calculated by compar-ing masked text to the original text)

Bio_ClinicalBERT	Verb Masking Ratio					
	**1.0**	**0.8**	**0.6**	**0.4**	**0.2**	**0.0**
**ROUGE-1**						
Generation Performance	96.48	97.38	97.97	98.54	99.08	99.51
Baseline	94.11	95.50	96.48	97.50	98.48	99.22
**ROUGE-2**						
Generation Performance	92.79	94.63	95.84	97.04	98.15	99.02
Baseline	88.53	91.26	93.18	95.19	97.14	98.61
**ROUGE-L**						
Generation Performance	96.37	97.31	97.92	98.51	99.07	99.50
Baseline	94.11	96.48	96.48	97.50	98.48	99.22
**BERTScore**						
Generation Performance	0.95	0.97	0.97	0.98	0.99	0.99
Baseline	0.82	0.86	0.89	0.92	0.95	0.98
**Invalid Prediction Rate**						
Generation Performance	0.31	0.31	0.32	0.32	0.33	0.37

Supplementary Table 11. Quantitative Comparisons of Verb Masking Ratios (The 'Baseline' was calculated by comparing masked text to the original text)

Bio_ClinicalBERT	Stopwords Masking Ratio					
	**1.0**	**0.8**	**0.6**	**0.4**	**0.2**	**0.0**
**ROUGE-1**						
Generation Performance	92.84	95.17	96.56	97.71	98.69	99.51
Baseline	81.52	85.53	89.04	92.54	96.04	99.22
**ROUGE-2**						
Generation Performance	84.64	89.41	92.53	95.05	97.24	99.02
Baseline	68.30	74.35	79.99	86.02	92.44	98.61
**ROUGE-L**						
Generation Performance	91.84	94.53	96.23	97.56	98.65	99.50
Baseline	81.52	85.53	89.04	92.54	96.04	99.22
**BERTScore**						
Generation Performance	0.89	0.93	0.95	0.97	0.98	0.99
Baseline	0.57	0.65	0.71	0.79	0.88	0.98
**Invalid Prediction Rate**						
Generation Performance	0.29	0.22	0.20	0.18	0.19	0.37

Supplementary Table 12. Quantitative Comparisons of Stopwords Masking Ratios (The 'Baseline' was calculated by comparing masked text to the original text)

**Bio_ ClinicalBERT**	**Masking Strategies**			
	**Noun Masking (0.4)**	**Stopwords Masking (0.2)**	**Verbs Masking (0.8)**	**Randomly Masking (0.1)**
**ROUGE-1**				
Generation Performance	97.62	98.69	97.62	98.28
Baseline	95.19	96.04	95.19	96.16
**ROUGE-2**				
Generation Performance	95.12	97.24	95.12	96.50
Baseline	90.82	92.44	90.82	92.68
**ROUGE-L**				
Generation Performance	97.56	98.65	97.56	98.25
Baseline	95.19	96.04	95.19	96.16
**BERTScore**				
Generation Performance	0.96	0.98	0.96	0.97
Baseline	0.86	0.88	0.86	0.88
**Invalid Prediction Rate**				
Generation Performance	0.32	0.19	0.32	0.25

Supplementary Table 13. Quantitative Comparison of Different Masking Strategies at a 0.04 Actual Masking Ratio (The 'Baseline' was calculated by comparing masked text to the original text)

**Bio_ClinicalBERT**	**Stopwords Masking (0.8)**	**Random Masking (0.4)**	**Nouns (0.5) and Stopwords (0.5)**	**Nouns (0.5), Verbs (0.5), Stopwords (0.8)**
**Actual Masking Ratio**	0.13	0.13	0.13	0.16
**ROUGE-1**				
Generation Performance	95.17	93.18	94.29	91.34
Baseline	85.53	85.61	85.98	82.47
**ROUGE-2**				
Generation Performance	89.41	86.50	88.34	83.08
Baseline	74.35	74.92	75.30	70.73
**ROUGE-L**				
Generation Performance	94.53	92.71	93.80	90.50
Baseline	85.53	85.61	85.98	82.47
**BERTScore**				
Generation Performance	0.93	0.90	0.91	0.87
Baseline	0.65	0.63	0.65	0.57
**Invalid Prediction Rate**				
Generation Performance	0.22	0.28	0.28	0.31

Supplementary Table 14. Quantitative Comparisons for Hybrid Masking (The Baseline was calculated by comparing masked text to the original text)
