# Supplementary figures and images for: Synthetic4Health: generating annotated synthetic clinical letters
Source: Front Digit Health. 2025 May 30;7:1497130. doi: 10.3389/fdgth.2025.1497130 (PMC12163008; doi:10.3389/fdgth.2025.1497130)

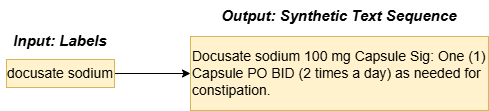

Supplement: Supplementary file 2 [file Datasheet2.zip › Sup Fig/Supplementary Figure S1.png]

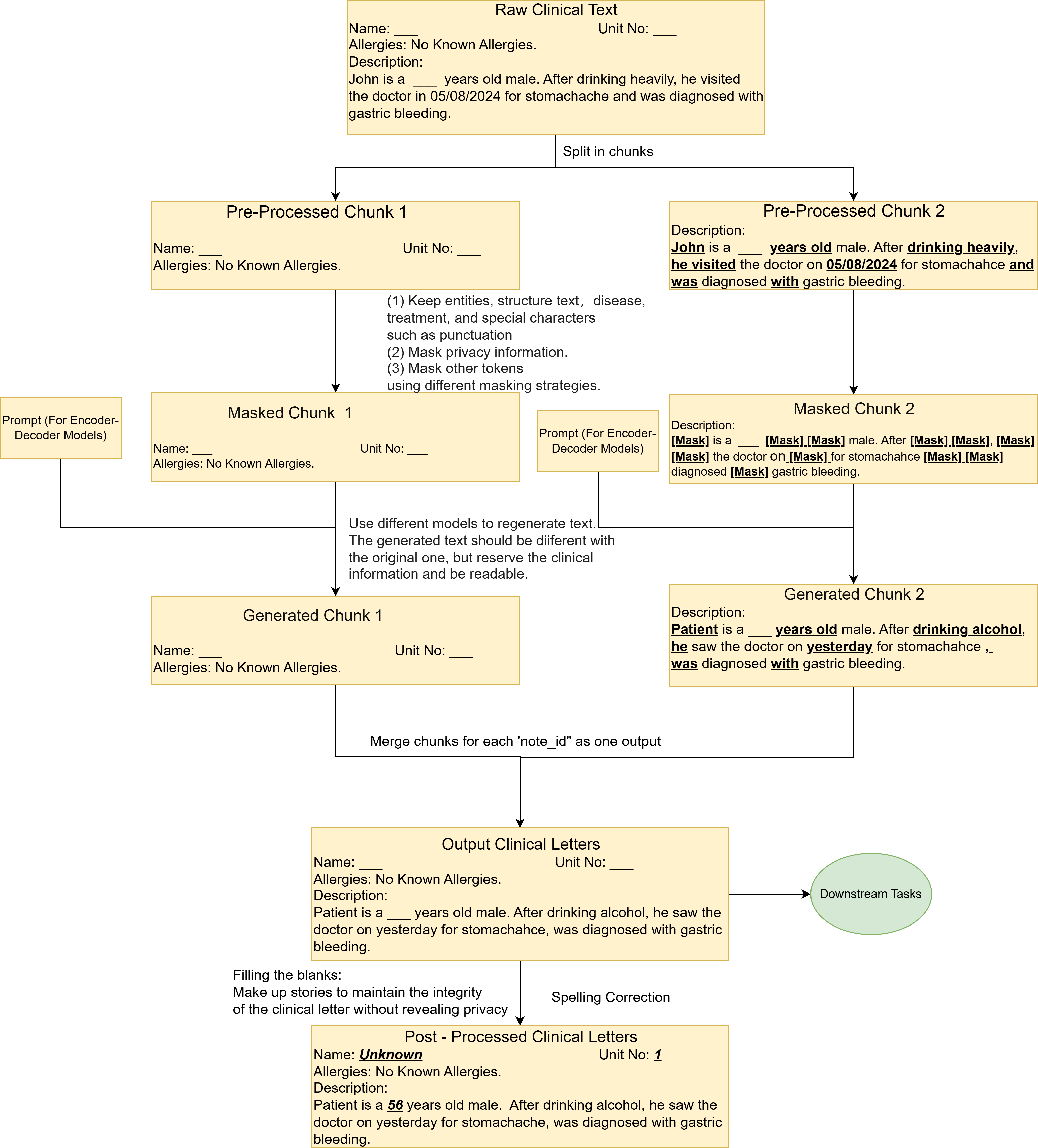

Supplement: Supplementary file 2 [file Datasheet2.zip › Sup Fig/Supplementary Figure S10.png]

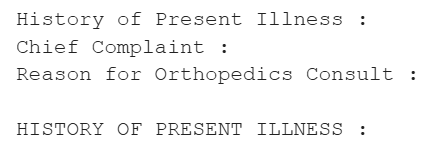

Supplement: Supplementary file 2 [file Datasheet2.zip › Sup Fig/Supplementary Figure S11.png]

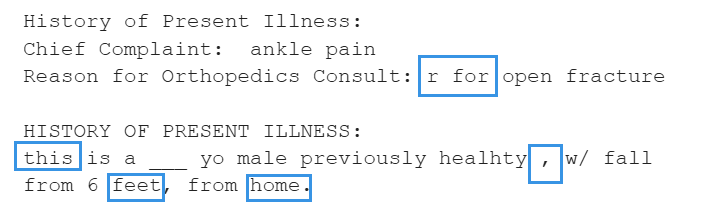

Supplement: Supplementary file 2 [file Datasheet2.zip › Sup Fig/Supplementary Figure S12.png]

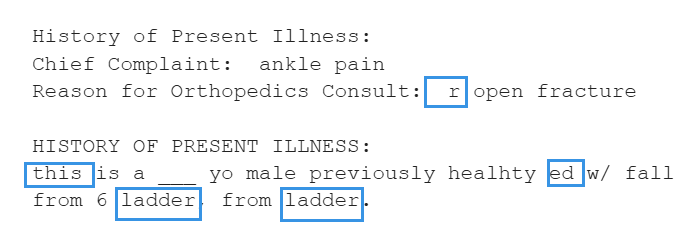

Supplement: Supplementary file 2 [file Datasheet2.zip › Sup Fig/Supplementary Figure S13.png]

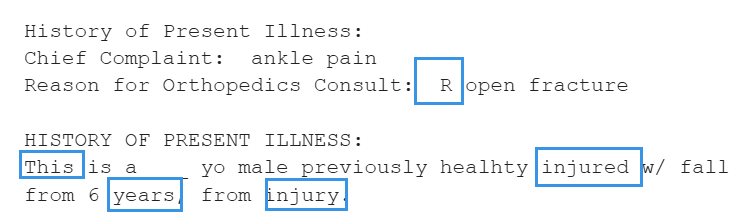

Supplement: Supplementary file 2 [file Datasheet2.zip › Sup Fig/Supplementary Figure S14.png]

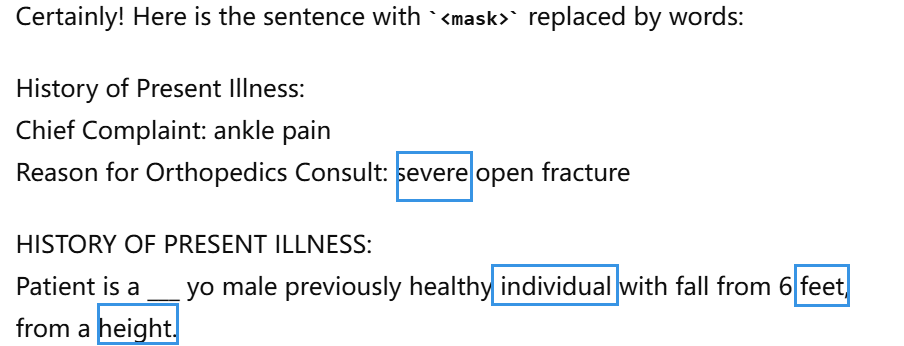

Supplement: Supplementary file 2 [file Datasheet2.zip › Sup Fig/Supplementary Figure S15.png]

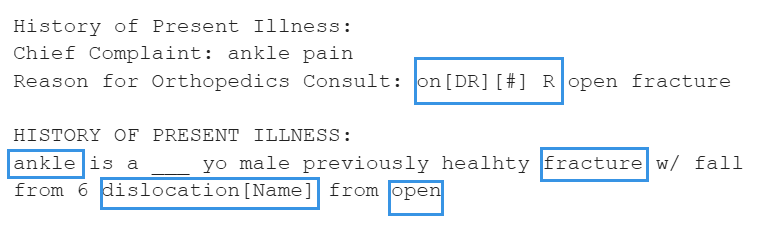

Supplement: Supplementary file 2 [file Datasheet2.zip › Sup Fig/Supplementary Figure S16.png]

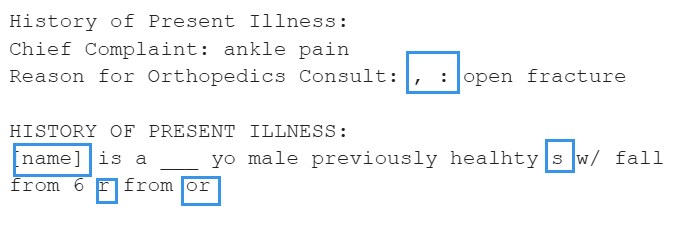

Supplement: Supplementary file 2 [file Datasheet2.zip › Sup Fig/Supplementary Figure S17.png]

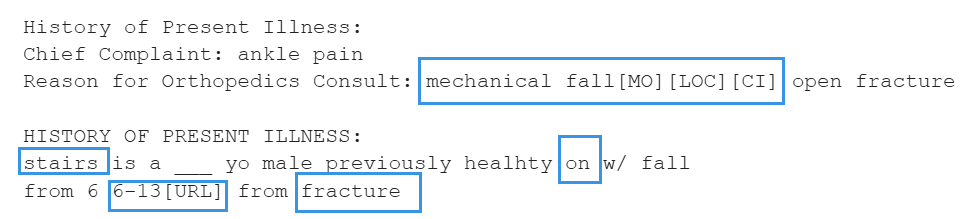

Supplement: Supplementary file 2 [file Datasheet2.zip › Sup Fig/Supplementary Figure S18.png]

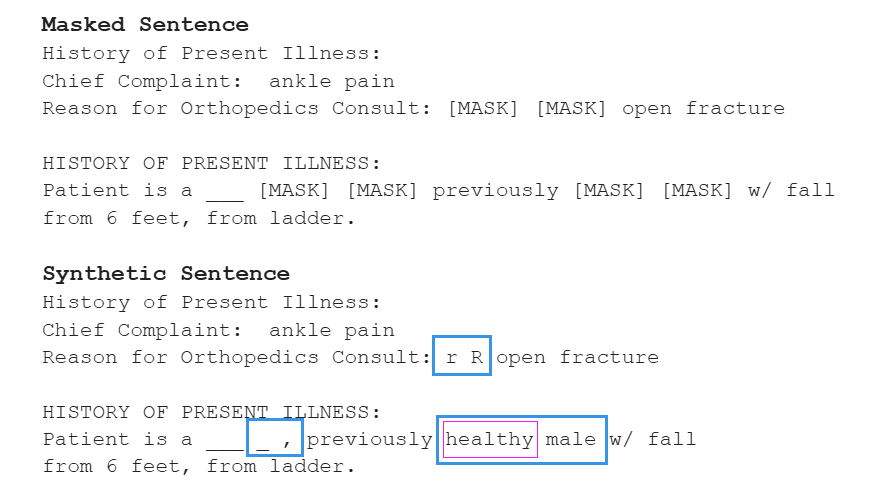

Supplement: Supplementary file 2 [file Datasheet2.zip › Sup Fig/Supplementary Figure S19.png]

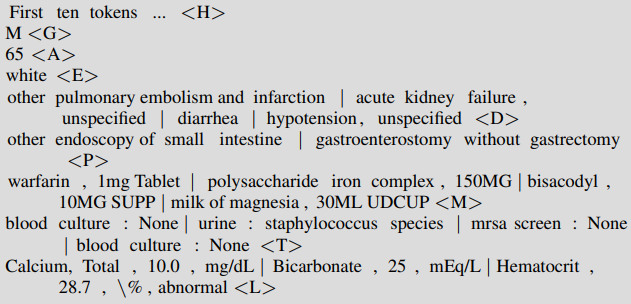

Supplement: Supplementary file 2 [file Datasheet2.zip › Sup Fig/Supplementary Figure S2.png]

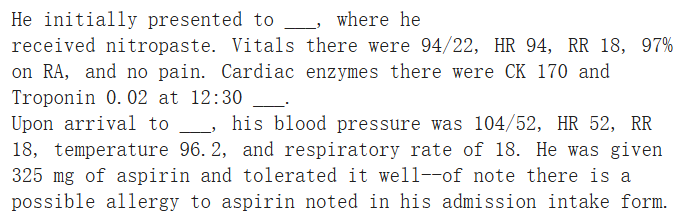

Supplement: Supplementary file 2 [file Datasheet2.zip › Sup Fig/Supplementary Figure S20.png]

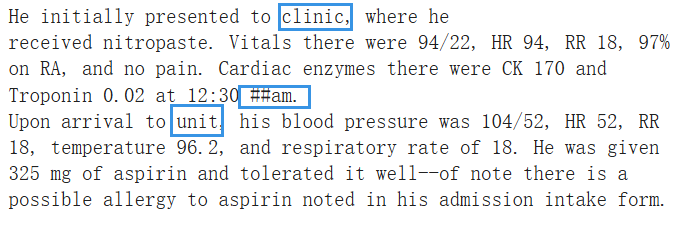

Supplement: Supplementary file 2 [file Datasheet2.zip › Sup Fig/Supplementary Figure S21.png]

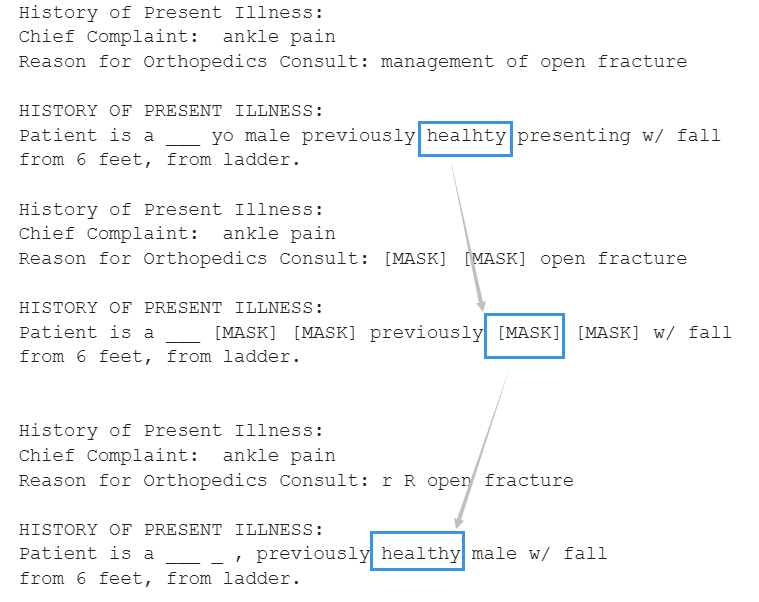

Supplement: Supplementary file 2 [file Datasheet2.zip › Sup Fig/Supplementary Figure S22.png]

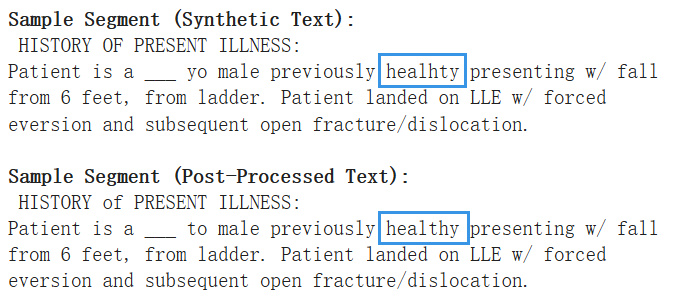

Supplement: Supplementary file 2 [file Datasheet2.zip › Sup Fig/Supplementary Figure S23.png]

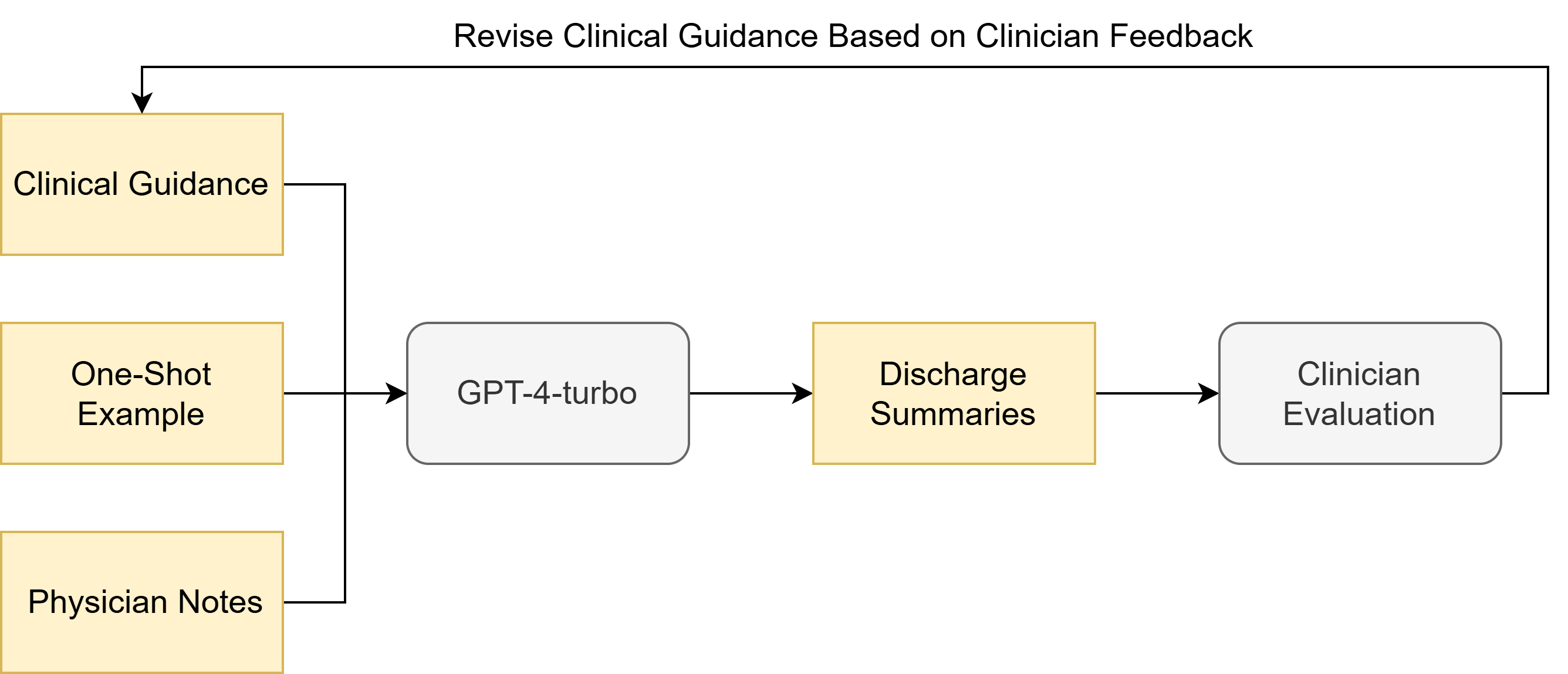

Supplement: Supplementary file 2 [file Datasheet2.zip › Sup Fig/Supplementary Figure S3.png]

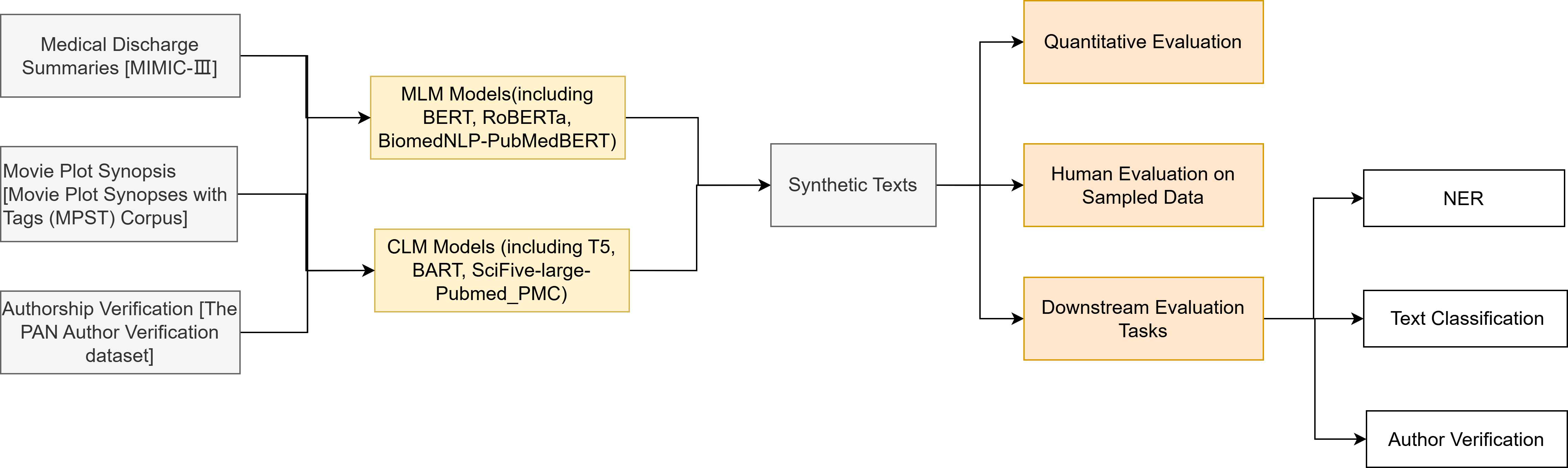

Supplement: Supplementary file 2 [file Datasheet2.zip › Sup Fig/Supplementary Figure S4.png]

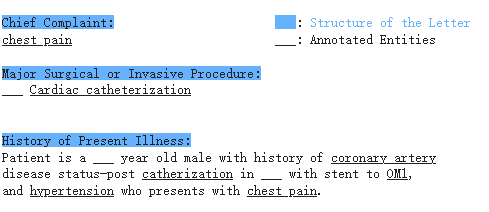

Supplement: Supplementary file 2 [file Datasheet2.zip › Sup Fig/Supplementary Figure S5.png]

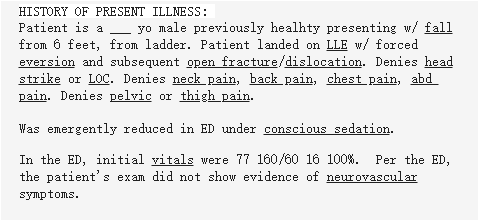

Supplement: Supplementary file 2 [file Datasheet2.zip › Sup Fig/Supplementary Figure S6.png]

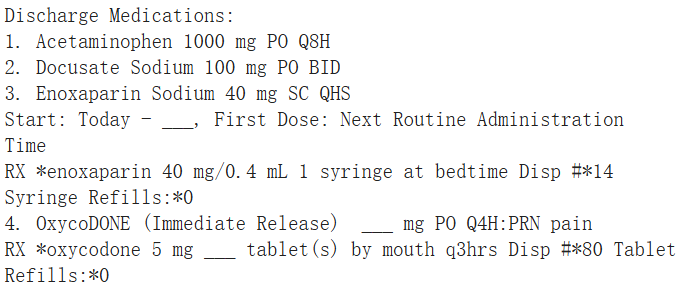

Supplement: Supplementary file 2 [file Datasheet2.zip › Sup Fig/Supplementary Figure S7.png]

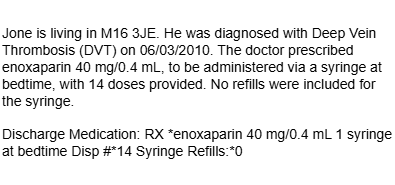

Supplement: Supplementary file 2 [file Datasheet2.zip › Sup Fig/Supplementary Figure S8.png]

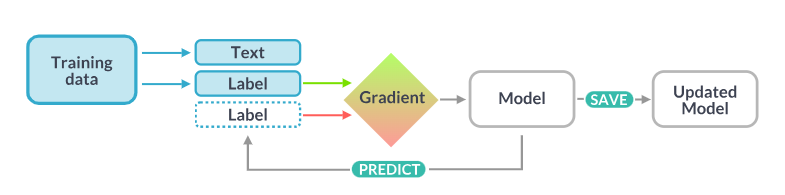

Supplement: Supplementary file 2 [file Datasheet2.zip › Sup Fig/Supplementary Figure S9.png]
